# Autophagy-Related Gene-Set Signals in the Genetic Architecture of Obsessive-Compulsive Disorder: A Hypothesis-Generating Link to Nicotinamide Adenine Dinucleotide-Associated Cellular Maintenance Biology

**DOI:** 10.7759/cureus.113327

**Published:** 2026-07-24

**Authors:** Ngo Cheung

**Affiliations:** 1 Psychiatry, Cheung Ngo Medical Centre, Hong Kong, HKG

**Keywords:** autophagy, cellular maintenance, genetic architecture, hypothesis-generating study, kegg pathway enrichment, mitophagy, nad+, obsessive-compulsive disorder, psychiatric genetics, twas

## Abstract

Obsessive-compulsive disorder (OCD) remains difficult to treat in a substantial minority of patients, despite the established first-line use of exposure and response prevention, selective serotonin reuptake inhibitors, and pharmacological augmentation. This treatment gap has encouraged renewed interest in biological axes that sit outside a narrow monoamine-centered model. In this study, we asked whether transcriptional genetic signals for OCD show enrichment in cellular maintenance pathways motivated by nicotinamide mononucleotide (NMN)-associated aging biology and whether any such pathways outperform a curated antidepressant-related gene comparator.

Using OCD transcriptome-wide association study (TWAS)-derived gene-level Z statistics and an absolute Z-score ranking framework, ten NMN-motivated Kyoto Encyclopedia of Genes and Genomes (KEGG) pathway expansions were compared against AntiDep_Genes. The strongest and most defensible finding in the OCD analysis was selective enrichment of KEGG_AUTOPHAGY_ANIMAL. Autophagy nominally outperformed the antidepressant comparator in the primary head-to-head test, with normalized enrichment score (NES) = 1.166, p = 0.041979, and difference in normalized enrichment score (dNES) = +0.2097. The signal persisted after removal of overlapping antidepressant-related genes, with cleaned NES = 1.1784 and p = 0.042479, and the autophagy set also showed a higher absolute Z-score distribution than AntiDep_Genes by Mann-Whitney U testing, p = 0.04717. Leading-edge overlap with the antidepressant comparator was minimal, limited to mechanistic target of rapamycin (MTOR).

The OCD autophagy signal was driven by a mixture of core autophagy machinery and upstream signaling genes, including MAPK3, MAP1LC3A, PPP2CB, MAP2K1, GORASP2, BAD, ATG2A, ATG10, MAPK8, and CALCOCO2. This pattern suggests a signaling-execution module rather than a simple enrichment of canonical ATG genes alone. The result is biologically consistent with recent OCD genetic findings implicating cortical and hippocampal excitatory neurons and D1/D2 striatal medium spiny neurons, which are cell types with high synaptic and metabolic demands. These findings are best viewed as hypothesis-generating. They do not support NMN, nicotinamide adenine dinucleotide (NAD+) precursors, or autophagy modulators as OCD treatments, but they do identify autophagy and mitochondrial quality-control biology as plausible targets for future functional and stratification studies.

The autophagy enrichment was nominally significant in the primary analysis but did not survive Benjamini-Hochberg false-discovery rate correction across the ten tested pathways (false discovery rate (FDR)-adjusted p = 0.41979); the findings should therefore be considered hypothesis-generating.

## Introduction

Obsessive-compulsive disorder (OCD) is a chronic psychiatric illness marked by intrusive obsessions, compulsions, avoidance, and often a long course of functional impairment. Although exposure and response prevention and selective serotonin reuptake inhibitors (SSRIs) are established first-line treatments, many patients do not remit, and a clinically important subset remains symptomatic after multiple trials. Contemporary reviews of OCD treatment describe cognitive behavioral therapy with exposure and response prevention and SSRIs as central first-line options, but also emphasize limits in access, tolerability, response, and durability [1]. For patients who do not respond adequately, augmentation with antipsychotics or other strategies can help some individuals, but the benefits are modest and uneven [2]. These clinical realities make OCD a good test case for asking whether disease biology may extend beyond the neurotransmitter systems most often targeted by current pharmacotherapy.

The serotonin and monoamine frameworks have been useful, especially because SSRIs and clomipramine can reduce OCD symptoms. Yet these frameworks do not fully explain why symptoms persist in many patients, why treatment response varies, or why OCD shares genetic liability with conditions that have different symptom profiles. A complementary view is that OCD risk may partly reflect altered cellular maintenance in vulnerable circuits. In this view, synapses and mitochondria are not passive downstream targets of psychiatric risk. They are active sites where genetic and environmental stress may converge. Neurons with long projections, high firing demands, or intense synaptic turnover require constant protein replacement, vesicle recycling, mitochondrial surveillance, and removal of damaged organelles. If these systems are genetically less resilient, circuit-level abnormalities could emerge even without a primary defect in any single neurotransmitter receptor.

Recent large-scale genetic work has made this kind of systems-level interpretation more plausible. Strom NI et al. [3] reported a large OCD genome-wide association study (GWAS) meta-analysis including 53,660 OCD cases and 2,044,417 controls, identifying 30 independent genome-wide significant loci and 249 potential effector genes, with 25 prioritized as likely causal candidates. Their tissue and cell-type analyses found OCD genetic signal enriched in excitatory neurons of the hippocampus and cortex, as well as D1 and D2 dopamine receptor-containing medium spiny neurons in the striatum. These findings fit long-standing cortico-striatal models of OCD, but they also point to broad neuronal maintenance demands. The same study reported strong genetic correlations with anxiety, depression, anorexia nervosa, Tourette syndrome, and post-traumatic stress disorder, as well as negative correlations with inflammatory bowel disease and body mass index. That pattern does not reduce OCD to any single pathway. It suggests a complex genetic architecture spanning neuronal, developmental, immune, and metabolic domains.

Autophagy is one candidate bridge across these domains. The term covers several related clearance and recycling programs, including macroautophagy, mitophagy, selective autophagy of protein aggregates, organelle turnover, and crosstalk with endolysosomal trafficking. In neurons, these processes are especially important because the cell body may be far from synaptic terminals, while presynaptic boutons face high demands for vesicle cycling, membrane exchange, and local protein turnover. The autophagy-endolysosomal system contributes to presynaptic protein turnover, synaptic vesicle quality control, and retrograde transport of degradative organelles [4]. Selective autophagy and mitophagy are also central to neuronal quality control, particularly in long-lived postmitotic cells that cannot dilute damaged proteins and organelles through cell division [5]. During development, neuronal and microglial autophagy participate in synaptic pruning and circuit refinement, making the pathway relevant not only to neurodegeneration but also to psychiatric circuit biology [6].

This study was also motivated by nicotinamide adenine dinucleotide (NAD+) and nicotinamide mononucleotide (NMN)-associated aging biology. In a re-analysis of GSE85718, Cheung [7] identified 35 genes whose age-associated expression changes were rescued by long-term NMN administration [8] in at least two of three metabolic tissues: skeletal muscle, liver, and white adipose tissue. No gene was rescued in all three tissues, implying tissue-specific rather than universal transcriptional rescue. The strongest mechanistic leads in that re-analysis were RAB11A, linked to endocytosis and vesicular trafficking, and CPT2, linked to fatty acid oxidation. MAP2K2, SLC13A5, MYO15, GALR1, TRP53INP2, and APBA2 provided additional links to stress signaling, metabolism, cytoskeletal dynamics, autophagy-related biology, and synaptic trafficking. These genes do not prove that NMN has psychiatric relevance. They do, however, motivate a broader question: do cellular resilience pathways highlighted by aging biology overlap with the genetic architecture of neuropsychiatric traits?

The connection between RAB11A and autophagy is particularly relevant. RAB11A-positive recycling endosomes can act as a platform for autophagosome formation, with WIPI2 recognizing coincident phosphatidylinositol 3-phosphate (PI3P) and RAB11A to direct autophagosome assembly [9]. A companion mechanistic summary [10] emphasized that coincidence detection between RAB11A and PI3P helps organize autophagosome biogenesis. Thus, the strongest NMN-associated trafficking signal and the autophagy pathway are not separate biological islands. They sit within the same cellular logistics system, one concerned with moving, sorting, recycling, and degrading membrane and protein cargo.

The present analysis therefore tested whether NMN-motivated Kyoto Encyclopedia of Genes and Genomes (KEGG) pathway expansions show enriched OCD transcriptome-wide association study (TWAS) signal relative to a curated antidepressant-related gene set. The goal was not to test NMN as a treatment for OCD. Nor was it to infer that any pathway enriched in OCD would be therapeutically beneficial if pharmacologically increased or decreased. The aim was narrower: to identify whether cellular maintenance pathways motivated by NMN-associated transcriptional biology capture a component of OCD genetic signal not already represented by antidepressant-related genes. Among ten tested pathways, the autophagy set emerged as the only nominally positive and biologically coherent OCD result.

The primary objective of this study was to test whether any of ten NMN-motivated KEGG pathway expansions showed enriched OCD TWAS association magnitude relative to a curated antidepressant-related gene comparator (AntiDep_Genes). Secondary objectives were to characterize the leading-edge genes driving any positive signal, perform robustness and specificity checks, including overlap removal, distributional tests, and signed-directionality analysis, and place the OCD result in the context of a broader head-to-head analysis across 11 traits. The study was designed as exploratory and hypothesis-generating; no therapeutic inferences regarding NMN, NAD+ precursors, or autophagy modulators were intended or are supported by the data.

## Materials and methods

**Figure 1:**
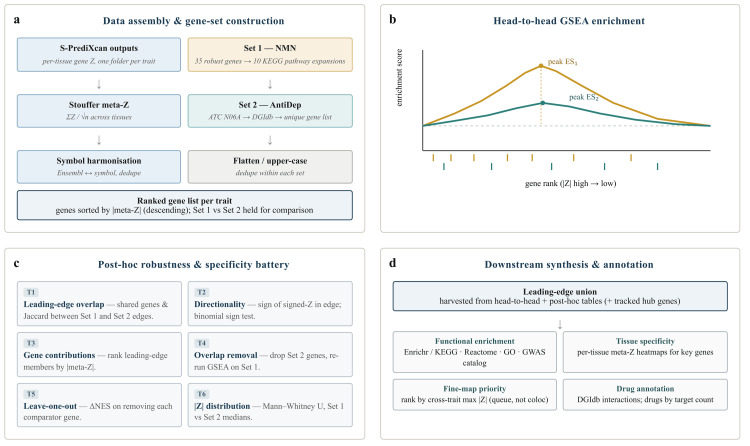
Overview of the gene-set proximity and head-to-head enrichment pipeline. (a) Trait-level S-PrediXcan statistics are combined across tissues by Stouffer’s method, harmonized to gene symbols, and ranked by |meta-Z|. In parallel, the NMN set and the antidepressant set, ATC class N06A mapped to targets via DGIdb, are flattened and de-duplicated, then held for comparison against each ranked trait list. (b) Weighted running-sum enrichment scores are computed for each set and evaluated against a competitive null of 2,000 size-matched random gene sets to yield NES and a permutation p-value. For each pair, the pipeline records dNES = NES₁ − NES₂ and a verdict, set1_beats_set2 if NES₁ > NES₂ and p₁ < 0.05. A hypergeometric over-representation analysis (|Z| > 3) is run in parallel. The curve shown is a schematic illustration of the running-sum statistic and does not depict analysis output. (c) Six post-hoc tests (T1-T6) probe the robustness and specificity of the head-to-head result: leading-edge overlap, directionality using a binomial sign test on signed Z values, per-gene contributions, overlap-removal re-testing, leave-one-out ΔNES, and Mann-Whitney comparison of |Z| distributions. A signed-rank re-analysis cross-checks the |Z|-based result, and Benjamini-Hochberg FDR correction is applied across the ten OCD pathway comparisons. (d) The union of leading-edge genes, plus tracked hub genes, is annotated through functional enrichment, per-tissue meta-Z specificity, a fine-mapping priority table ranked by cross-trait maximum |Z|, and DGIdb drug annotation. Cross-trait aggregation counts recurrent leading-edge genes and nominal wins per set pair; all downstream steps are exploratory annotations only. TWAS: Transcriptome-wide association study; GSEA: Gene-set enrichment analysis; NES: Normalized enrichment score; ORA: Over-representation analysis; FDR: False discovery rate; ATC: Anatomical Therapeutic Chemical classification; DGIdb: Drug-Gene Interaction Database. Credit: Ngo Cheung. Figure prepared using Microsoft PowerPoint. No AI tools were used.

This analysis used gene-level TWAS-style meta-Z statistics derived from S-PrediXcan outputs (Figure 1). For each trait folder, per-tissue gene Z statistics were loaded from tabular files, gene identifiers were harmonized to uppercase gene symbols where possible, and a cross-tissue meta-Z was calculated for each gene using Stouffer’s method. The OCD gene-level statistics used here were interpreted in the context of the large OCD genetic analysis by Strom NI et al. [3], which identified genome-wide loci, prioritized effector genes, and reported brain tissue and cell-type enrichments relevant to OCD.

The Stouffer meta-Z combination, modified GSEA-style running-sum calculation, permutation testing, overlap-removal retesting, and leave-one-out analyses were implemented in a custom computational workflow. The starting NMN set was the 35-gene set from the Cheung N [7] re-analysis of GSE85718, with the underlying NMN study reported by Mills KF et al. [8]. Functional enrichment used the Enrichr KEGG 2021 Human and Reactome 2022 libraries.

The NMN-motivated gene sets began with 35 robust genes from the Cheung [7] re-analysis of GSE85718. These 35 genes were not used directly as a single small set. Instead, they were mapped to standard KEGG pathways that represented their main biological themes. Ten KEGG pathway sets were included: endocytosis, fatty acid degradation, MAPK signaling, Ras signaling, PPAR signaling, neuroactive ligand-receptor interaction, regulation of actin cytoskeleton, citrate cycle/TCA cycle, autophagy animal, and synaptic vesicle cycle. These pathway expansions were intended to capture broader biological programs related to the robust NMN-associated genes, especially vesicular trafficking, mitochondrial substrate use, stress signaling, and cellular quality control.

For the primary analysis, the 35 robust genes served as anchors for pathway expansion rather than as a single 35-gene test set. Gene symbols were flattened and de-duplicated within each expanded set. The complete anchor-to-pathway mapping, full pathway membership lists, and any manual curation decisions were not included in the available analysis record and should accompany the computational materials.

The comparator, AntiDep_Genes, was built from antidepressant-related drug-gene information. Antidepressant drugs under ATC group N06A were collected from the WHO ATC/DDD index workflow, drug names were queried against DGIdb-derived interaction results, and the resulting gene lists were flattened into a unique antidepressant-related gene set. This comparator was used to represent a broad pharmacological gene space related to existing antidepressant treatment rather than a narrowly monoaminergic list. Because some genes occur in broad signaling and neuroactive pathways as well as drug interaction databases, overlap between the NMN-motivated sets and AntiDep_Genes was measured and tested explicitly.

The final comparator was the de-duplicated union of genes returned by that workflow. The available analysis record does not specify the complete ATC N06A drug query list, the DGIdb interaction types retained, any interaction-score threshold, or the final unique-gene count; no unreported filter or count is inferred here.

Gene symbols were converted to uppercase where possible. Only genes with a matching gene-level meta-Z were eligible for the ranked analyses, so pathway members absent from the TWAS universe could not contribute. A separate alias or retired-symbol crosswalk was not documented in the available workflow.

The primary enrichment framework used a modified GSEA-style ranking of all genes by absolute meta-Z, so that both positive and negative predicted-expression associations could contribute to enrichment. This choice was deliberate. The goal was to test whether pathway genes carried stronger aggregate OCD TWAS signal than comparator genes, not whether the pathway showed a single direction of predicted expression. For each gene set, an enrichment score and NES were calculated. Competitive permutation p-values were generated using random gene sets of the same size, with 2,000 permutations in the main analysis. A head-to-head comparison was then made for each NMN-motivated KEGG pathway against AntiDep_Genes. The difference in NES was recorded as dNES. A pathway was labeled as beating the comparator when its NES exceeded the comparator NES and its own GSEA p-value was below 0.05.

The permutation null was competitive and size-matched; no additional gene-level stratification or blocking was documented. For the ten primary OCD pathway comparisons, Benjamini-Hochberg FDR correction was applied to the ten pathway-level p-values (p1).

Several robustness and specificity checks were applied to the OCD result. First, genes overlapping between each NMN-motivated set and AntiDep_Genes were removed from the NMN set, and the enrichment test was repeated. This tested whether any signal was simply driven by shared antidepressant-related genes. Second, leading-edge overlap was quantified with overlap counts and Jaccard indices. Third, the absolute Z-score distributions of each NMN-motivated set and AntiDep_Genes were compared using the Mann-Whitney U test. Fourth, directionality was examined using signed meta-Z values among leading-edge genes, with a binomial sign test comparing positive and negative Z counts. This tested a simple repression model, including the possibility of a Polycomb-like negative-Z skew. Fifth, leave-one-out analysis was used to assess whether the comparator enrichment was driven by a few high-Z genes.

Post-hoc follow-up analyses were used only for interpretation. A 795-gene leading-edge union was assembled from the top leading-edge genes across the NMN-motivated and antidepressant sets in OCD, including manually tracked hub genes. Enrichr was used to assess broad functional enrichment of this union. Tissue-level specificity was explored for selected genes across available brain S-PrediXcan tissues. A “colocalization/fine-mapping priority” table ranked candidate genes by maximum absolute Z across the included trait context, but this was a prioritization exercise and not a formal variant-level colocalization analysis. DGIdb interaction output was used to identify drugs with many interactions among leading-edge genes, but this was treated as an exploratory annotation only. No therapeutic inference was made from the DGIdb screen.

All reported p-values from the pathway head-to-head tests are nominal unless explicitly described as adjusted. The primary OCD multiplicity adjustment was applied across the ten pathway comparisons, whereas post-hoc overlap, distributional, directionality, and leave-one-out analyses were treated as supportive internal checks rather than independent confirmatory tests. The analysis tested multiple pathways and traits, so modest p-values were interpreted cautiously. The Enrichr follow-up used adjusted p-values within its own enrichment framework, but those results were expected to be broad because the input union was assembled from pathway-derived leading-edge genes. The work used aggregate gene-level results and public or de-identified resources. No new individual-level human subject data were analyzed.

Analysis code, the ten NMN-motivated pathway gene lists, and the AntiDep_Genes comparator list will be made available from the corresponding author upon reasonable request.

## Results

Primary OCD finding: selective enrichment of autophagy-related genes

Among the ten NMN-motivated KEGG pathway sets, KEGG_AUTOPHAGY_ANIMAL was the only pathway that nominally outperformed AntiDep_Genes in OCD. In the primary head-to-head analysis, the autophagy set had NES = 1.1661 and p = 0.041979, while AntiDep_Genes had NES = 0.9564 and p = 0.677661. The resulting dNES was +0.2097, and the pathway was labeled “set1_beats_set2” (Table 1). In practical terms, this means that autophagy-related genes, as a pathway group, were more concentrated toward the high absolute OCD TWAS signal ranks than the antidepressant-related comparator.

**Table 1:** OCD head-to-head pathway results for NMN-motivated KEGG sets versus AntiDep_Genes. OCD: Obsessive-compulsive disorder; NMN: Nicotinamide mononucleotide; KEGG: Kyoto Encyclopedia of Genes and Genomes; AntiDep_Genes: Antidepressant-related gene comparator.

NMN pathway set	NES1	p1	NES2	p2	dNES	Verdict
KEGG_AUTOPHAGY_ANIMAL	1.1661	0.041979	0.9564	0.677661	0.2097	set1_beats_set2
KEGG_RAS_SIGNALING_PATHWAY	1.1167	0.10095	0.9564	0.677661	0.1603	Not better
KEGG_MAPK_SIGNALING_PATHWAY	1.0893	0.151924	0.9564	0.677661	0.1329	Not better
KEGG_ENDOCYTOSIS	1.0197	0.3998	0.9564	0.677661	0.0633	Not better
KEGG_SYNAPTIC_VESICLE_CYCLE	0.9505	0.627686	0.9564	0.677661	-0.0059	Not better
KEGG_FATTY_ACID_DEGRADATION	0.9282	0.66017	0.9564	0.677661	-0.0282	Not better
KEGG_REGULATION_OF_ACTIN_CYTOSKELETON	0.9014	0.870065	0.9564	0.677661	-0.055	Not better
KEGG_NEUROACTIVE_LIGAND_RECEPTOR_INTERACTION	0.8944	0.897551	0.9564	0.677661	-0.062	Not better
KEGG_CITRATE_CYCLE_TCA_CYCLE	0.8236	0.815092	0.9564	0.677661	-0.1328	Not better
KEGG_PPAR_SIGNALING_PATHWAY	0.7535	0.957021	0.9564	0.677661	-0.2029	Not better

Because ten NMN-motivated pathways were evaluated in parallel against the same comparator, Benjamini-Hochberg FDR correction was applied across the ten pathway-level p1 values in Table 1. The autophagy p-value was nominally significant (p = 0.041979), but the FDR-adjusted p-value was 0.41979 and did not meet FDR < 0.05. All p-values reported in this section are therefore interpreted as nominal unless explicitly labeled adjusted; the autophagy enrichment should be viewed as a candidate signal requiring independent replication rather than a definitive finding.

The nominal signal was modest, but it persisted in the most important specificity check. Leading-edge overlap between KEGG_AUTOPHAGY_ANIMAL and AntiDep_Genes was minimal. The only shared leading-edge gene was MTOR, with an overlap count of 1 and a Jaccard index of 0.0082. After removing genes overlapping with AntiDep_Genes, the autophagy signal did not disappear. It slightly strengthened, with cleaned NES = 1.1784 and p = 0.042479. At the nominal level, this argues against the simplest artifact: that the autophagy result was merely tagging known antidepressant-related neurotransmitter or pharmacology genes.

A distributional check supported the same interpretation. KEGG_AUTOPHAGY_ANIMAL had a higher median absolute Z-score than AntiDep_Genes, with Mann-Whitney U p = 0.04717. The median absolute Z was 1.3478 for the autophagy set and 1.1568 for AntiDep_Genes. This result is again modest, and it should not be overinterpreted as a large effect. Still, it points in the same direction as the GSEA result: in OCD, the autophagy pathway carried a slightly stronger aggregate TWAS signal than the antidepressant comparator.

The cleaned NES of 1.1784 and the Mann-Whitney result were also modest; these analyses are best regarded as supportive internal checks rather than independent confirmations, particularly because the primary autophagy result did not survive correction across the ten pathways.

Leading-Edge Composition: A Signaling-Execution Module

The top autophagy leading-edge genes were not limited to canonical autophagy machinery. Instead, they formed a mixed signaling and execution module. The strongest drivers included MAPK3, with Z = +6.064; MAP1LC3A, Z = +5.960; PPP2CB, Z = -5.938; MAP2K1, Z = -5.646; GORASP2, Z = +5.506; BAD, Z = +5.442; ATG2A, Z = +5.293; ATG10, Z = -5.236; MAPK8, Z = +4.695; and CALCOCO2, Z = +4.578. The Table 2 subset also included PPP2CA, MAP2K2, CTSD, DAPK2, and HRAS; the broader leading edge additionally included VPS33A, RPS6KB2, RAB7A, VPS39, PRKACA, TANK, MAPK1, AKT3, PRKACB, and PIK3R1.

**Table 2:** Principal OCD leading-edge genes for KEGG_AUTOPHAGY_ANIMAL and AntiDep_Genes. OCD: Obsessive-compulsive disorder; KEGG: Kyoto Encyclopedia of Genes and Genomes; AntiDep_Genes: Antidepressant-related gene comparator; NMN: Nicotinamide mononucleotide.

Group	Gene set	Gene	Z	Absolute Z	Rank
NMN	KEGG_AUTOPHAGY_ANIMAL	MAPK3	6.064	6.064	153
NMN	KEGG_AUTOPHAGY_ANIMAL	MAP1LC3A	5.96	5.96	167
NMN	KEGG_AUTOPHAGY_ANIMAL	PPP2CB	-5.938	5.938	173
NMN	KEGG_AUTOPHAGY_ANIMAL	MAP2K1	-5.646	5.646	220
NMN	KEGG_AUTOPHAGY_ANIMAL	GORASP2	5.506	5.506	247
NMN	KEGG_AUTOPHAGY_ANIMAL	BAD	5.442	5.442	265
NMN	KEGG_AUTOPHAGY_ANIMAL	ATG2A	5.293	5.293	297
NMN	KEGG_AUTOPHAGY_ANIMAL	ATG10	-5.236	5.236	324
NMN	KEGG_AUTOPHAGY_ANIMAL	MAPK8	4.695	4.695	496
NMN	KEGG_AUTOPHAGY_ANIMAL	CALCOCO2	4.578	4.578	545
NMN	KEGG_AUTOPHAGY_ANIMAL	PPP2CA	4.451	4.451	602
NMN	KEGG_AUTOPHAGY_ANIMAL	MAP2K2	-4.402	4.402	629
NMN	KEGG_AUTOPHAGY_ANIMAL	CTSD	-4.379	4.379	640
NMN	KEGG_AUTOPHAGY_ANIMAL	DAPK2	4.359	4.359	651
NMN	KEGG_AUTOPHAGY_ANIMAL	HRAS	-4.026	4.026	845
AntiDep	AntiDep_Genes	ANK3	9.123	9.123	12
AntiDep	AntiDep_Genes	METTL21A	-5.244	5.244	320
AntiDep	AntiDep_Genes	RABEP1	-4.709	4.709	491
AntiDep	AntiDep_Genes	KCNH2	4.606	4.606	531
AntiDep	AntiDep_Genes	GMNN	4.119	4.119	770
AntiDep	AntiDep_Genes	CBX1	4.014	4.014	863
AntiDep	AntiDep_Genes	GNB3	-3.951	3.951	921
AntiDep	AntiDep_Genes	EHMT2	3.803	3.803	1053
AntiDep	AntiDep_Genes	MPHOSPH8	3.54	3.54	1271
AntiDep	AntiDep_Genes	MC1R	-3.532	3.532	1281
AntiDep	AntiDep_Genes	CHRM3	3.531	3.531	1283
AntiDep	AntiDep_Genes	DTNBP1	-3.505	3.505	1315
AntiDep	AntiDep_Genes	PPM1A	-3.369	3.369	1469
AntiDep	AntiDep_Genes	ERCC8	3.291	3.291	1554
AntiDep	AntiDep_Genes	SLC39A14	-3.224	3.224	1635

This composition matters. MAP1LC3A, ATG2A, ATG10, CALCOCO2, RAB7A, VPS33A, and VPS39 are close to autophagosome formation, cargo recognition, vesicle maturation, or autolysosomal trafficking. MAPK3, MAP2K1, MAPK8, MAP2K2, HRAS, AKT3, PRKACA, and PIK3R1 sit upstream in growth factor, stress-response, and kinase signaling networks that influence when and how autophagy is engaged. BAD and DAPK2 connect autophagy to cell stress and apoptosis-related signaling. PPP2CB and PPP2CA suggest phosphatase-mediated fine-tuning of signaling and autophagic flux. The result therefore does not look like a narrow “ATG-only” signal. It looks more like genetic association with the decision machinery that regulates autophagy under cellular stress.

In OCD circuit terms, this is biologically plausible. Strom et al. [3] found OCD genetic enrichment in cortical and hippocampal excitatory neurons and in striatal D1/D2 medium spiny neurons. These cell types are not interchangeable, but they share high demands for synaptic maintenance, calcium handling, local protein turnover, and mitochondrial quality control. Autophagy and the endolysosomal system are known to support presynaptic function and synaptic protein turnover, especially at axonal boutons where vesicle recycling and local protein damage create a need for efficient clearance [4]. Mitophagy and selective autophagy also provide a means to remove damaged mitochondria and other cargo in neurons with long processes and high energy requirements [5].

Negative findings and specificity

The OCD result was selective. None of the other NMN-motivated KEGG pathway sets met the nominal head-to-head criterion in OCD. KEGG_RAS_SIGNALING_PATHWAY had NES = 1.1167 and p = 0.100950; KEGG_MAPK_SIGNALING_PATHWAY had NES = 1.0893 and p = 0.151924; KEGG_ENDOCYTOSIS had NES = 1.0197 and p = 0.399800. The remaining pathways, including synaptic vesicle cycle, fatty acid degradation, actin cytoskeleton regulation, neuroactive ligand-receptor interaction, citrate cycle/TCA cycle, and PPAR signaling, did not meet the head-to-head significance criterion in OCD. These negative results help narrow the interpretation. The OCD finding was not simply “all NMN-motivated biology” or “all broad KEGG pathways.” It was most clearly autophagy.

The directionality analysis also narrowed the interpretation. No gene set showed a significant positive or negative sign imbalance among leading-edge genes. For KEGG_AUTOPHAGY_ANIMAL, the leading edge contained 45 genes, with 23 positive and 22 negative signed Z values, mean signed Z = +0.2199, and sign-test p = 1.0000. AntiDep_Genes had 78 leading-edge genes, with 33 positive and 45 negative values, mean signed Z = -0.2916, and sign-test p = 0.21268. Thus, the autophagy signal cannot be described as a simple repression signature. The signed re-analysis supported this caution. Under signed ranking, KEGG_AUTOPHAGY_ANIMAL had NES = +1.0085 and p = 0.234883, while AntiDep_Genes had NES = -1.0273 and p = 0.173913; the comparison was not significant. The absolute-ranking result therefore reflects stronger aggregate association magnitude, not a uniform predicted upregulation or downregulation pattern. These checks are summarized in Table 3.

**Table 3:** OCD robustness, overlap, distributional, and signed-ranking checks for KEGG_AUTOPHAGY_ANIMAL. OCD: Obsessive-compulsive disorder; KEGG: Kyoto Encyclopedia of Genes and Genomes; AntiDep_Genes: Antidepressant-related gene comparator; NES: Normalized enrichment score; dNES: Difference in normalized enrichment score; Z: Z-score; MTOR: Mechanistic target of rapamycin; FDR: False discovery rate.

Analysis	KEGG_AUTOPHAGY_ANIMAL result	Comparator or overlap result	p-value or statistic	Interpretation
Primary head-to-head	NES = 1.1661	AntiDep_Genes NES = 0.9564	p1 = 0.041979; dNES = 0.2097	Autophagy nominally beats comparator
Leading-edge overlap	45 leading-edge genes	1 shared gene with AntiDep_Genes: MTOR	Jaccard = 0.0082	Minimal overlap
Overlap-removal retest	Cleaned NES = 1.1784	Comparator NES = 0.9591	cleaned p = 0.042479	Signal persists after overlap removal
Absolute Z distribution	Median absolute Z = 1.3478	AntiDep_Genes median absolute Z = 1.1568	Mann-Whitney U p = 0.04717	Higher absolute signal
Signed leading-edge directionality	23 positive, 22 negative; mean Z = 0.2199	No sign skew	Sign-test p = 1.0000	No directional imbalance
Signed-ranking re-analysis	NES1 = 1.0085	NES2 = -1.0273	p1 = 0.234883; p2 = 0.173913	No significant directional win

The antidepressant comparator itself was influenced by several high absolute Z genes, including ANK3, METTL21A, RABEP1, KCNH2, GMNN, CBX1, GNB3, EHMT2, MPHOSPH8, MC1R, CHRM3, DTNBP1, PPM1A, ERCC8, SLC39A14, C1QBP, ATP4A, GSK3B, CYP2D6, ACE, ANO2, MTOR, UST, HTR1B, and MIEF2. Leave-one-out analysis showed that removing ANK3 caused the largest reduction in AntiDep_Genes NES, from 0.9591 to 0.9349, with dNES = -0.0242. Other leave-one-out effects were smaller. This does not invalidate the comparator, but it shows that the antidepressant-related set was heterogeneous and partly driven by a small number of strong genes.

Post-hoc functional and tissue-level context

The OCD follow-up assembled a leading-edge union of 795 unique genes. For the two focal comparator sets, the follow-up used 30-gene prioritized leading-edge subsets from AntiDep_Genes and KEGG_AUTOPHAGY_ANIMAL; these should be distinguished from the full primary leading-edge counts of 78 and 45 genes, respectively. The broader union also included leading-edge genes from the other NMN-motivated pathway sets. All tracked hub genes were present in the union: GABRA6, RPTOR, MAPK3, GABRB2, GNB3, DRD2, GSK3B, ANK3, CYP2D6, CHRM5, HTR6, CACNA1B, MAPK1, SF3B1, FYN, LIMK1, CTNNB1, and GLS2.

Functional enrichment of this union returned 2,134 significant terms at adjusted p < 0.05 among 8,107 tested terms. The strongest terms were broad and expected, given the way the union was constructed. These included Reactome Signal Transduction, adjusted p = 3.18E-119; KEGG Endocytosis, adjusted p = 2.20E-109; KEGG Regulation of actin cytoskeleton, adjusted p = 1.11E-106; KEGG MAPK signaling pathway, adjusted p = 6.36E-99; KEGG Neuroactive ligand-receptor interaction, adjusted p = 1.91E-95; KEGG Ras signaling pathway, adjusted p = 2.15E-88; KEGG Autophagy, adjusted p = 1.55E-82; KEGG PPAR signaling pathway, adjusted p = 1.14E-67; KEGG Synaptic vesicle cycle, adjusted p = 2.43E-63; and Reactome GPCR Downstream Signaling, adjusted p = 2.57E-62 (Table 4). These results should be treated as descriptive confirmation of pathway content rather than independent discovery, because the input set was assembled from pathway-derived leading edges.

**Table 4:** Functional enrichment of the 795-gene leading-edge union: top terms. KEGG: Kyoto Encyclopedia of Genes and Genomes; MAPK: Mitogen-activated protein kinase; PPAR: Peroxisome proliferator-activated receptor; GPCR: G protein-coupled receptor.

Library	Term	Adjusted p-value	Overlap
Reactome 2022	Signal Transduction R-HSA-162582	3.18E-119	358/2465
KEGG 2021 Human	Endocytosis	2.20E-109	128/252
KEGG 2021 Human	Regulation of actin cytoskeleton	1.11E-106	119/218
KEGG 2021 Human	MAPK signaling pathway	6.36E-99	128/294
KEGG 2021 Human	Neuroactive ligand-receptor interaction	1.91E-95	133/341
KEGG 2021 Human	Ras signaling pathway	2.15E-88	109/232
KEGG 2021 Human	Autophagy	1.55E-82	85/137
KEGG 2021 Human	PPAR signaling pathway	1.14E-67	59/74
KEGG 2021 Human	Synaptic vesicle cycle	2.43E-63	58/78
Reactome 2022	GPCR Downstream Signaling R-HSA-388396	2.57E-62	138/619

The tissue-level follow-up included 333 rows across 74 genes, six brain tissues, and one trait context. ANK3 showed the strongest positive tissue-level signal, with Z = +4.0377 across several brain regions, including the frontal cortex, nucleus accumbens, caudate, anterior cingulate cortex, and hippocampus. GLS2 showed a repeated negative signal, Z = -3.4822, across multiple regions, including the nucleus accumbens, caudate, frontal cortex, amygdala, and hippocampus. Other notable tissue-level signals included ACO2 in the caudate and nucleus accumbens, HTR6 across the hippocampus, caudate, anterior cingulate, and frontal cortex, MAPK3 across the frontal cortex, amygdala, hippocampus, and nucleus accumbens, DRD2 in the nucleus accumbens, and MAPK1 in the caudate and nucleus accumbens. These observations are consistent with cortico-striatal and limbic relevance but are not a substitute for cell-type-specific functional validation.

The prioritization table ranked 795 candidate genes by maximum absolute Z. The highest-priority genes in this descriptive ranking were ANK3, SNF8, GLS2, CHRNB1, LAMTOR3, VPS45, LYNX1, WASHC3, PPP1CB, MAPK3, MAP1LC3A, PPP2CB, C3, VPS4A, VPS28, MAP2K1, GORASP2, PFN1, BAD, ACO2, HTR6, ATG2A, METTL21A, ATG10, and TAC3. This list includes both autophagy-related and non-autophagy genes. It should be interpreted as a queue for future fine mapping and functional work, not as proof of causality.

The DGIdb annotation screen found 16,687 drug-gene interactions, 7,568 distinct drugs, and 480 genes hit among the leading-edge union. The top drugs by number of distinct leading-edge targets shown in the top-count rows of Table 5 were sorafenib, PF-562271, cenisertib, NVP-TAE684, ilorasertib, RG-1530, dasatinib anhydrous, dovitinib, clozapine, and doxorubicin hydrochloride. Additional high-count DGIdb entries not shown among the Table 5 top-count rows included CYC-116, haloperidol decanoate, olanzapine, cisplatin, tamatinib, SP-600125, linifanib, tozasertib, aspirin, celecoxib, cyclophosphamide anhydrous, methotrexate, doxepin hydrochloride, gefitinib, and PD-0166285. This screen is not a therapeutic recommendation. It is a descriptive annotation of database-recorded drug-gene relationships and carries no therapeutic implication for OCD. Many of the highest-ranking compounds are oncology or kinase agents with toxicity profiles that make them unsuitable for psychiatric repurposing without a very different evidentiary basis. The value of this screen is mainly that it highlights druggable biology and shows that some existing psychiatric agents, including lithium, olanzapine, carbamazepine, and lamotrigine, intersect with genes in the broader leading-edge union.

**Table 5:** Exploratory DGIdb drug ranking and selected psychotropic overlap. DGIdb: Drug-Gene Interaction Database.

Category	Drug	Number of targets	Mean score	Representative targets
Top DGIdb target count	Sorafenib	38	0.042	ABCB1; ATXN2; BRAF; CBL; CSF1R; CTNNB1; DRD2; EGFR; EPHA2; FLT3
Top DGIdb target count	PF-562271	35	0.026	ACSL1; ACSL3; CAMKK2; DAPK3; EPHA2; FLT3; FLT4; FYN; GRK5; GSK3B
Top DGIdb target count	Cenisertib	31	0.013	AKT1; CSF1R; DAPK3; DRD2; EGFR; ERBB4; FLT3; FLT4; FYN; INSR
Top DGIdb target count	NVP-TAE684	29	0.015	CSF1R; DAPK3; EGFR; ERBB4; FLT3; FLT4; FYN; GRK5; INSR; IRAK4
Top DGIdb target count	Ilorasertib	28	0.012	CSF1R; DRD2; EGFR; EPHA2; ERBB4; FLT3; FLT4; FYN; INSR; IRAK4
Top DGIdb target count	RG-1530	27	0.014	CSF1R; DRD2; EGFR; EPHA2; ERBB4; FLT3; FLT4; FYN; GRK5; GSK3B
Top DGIdb target count	Dasatinib anhydrous	26	0.05	ABCB1; ABL2; BRAF; CBL; CRKL; CSF1R; EPHA2; ERBB4; FYN; GMNN
Top DGIdb target count	Dovitinib	26	0.024	CAMKK2; CFLAR; CSF1R; DRD2; FGFR4; FLT3; FLT4; FYN; INSR; LIMK1
Top DGIdb target count	Clozapine	25	0.047	A1BG; A2M; ABCF1; ALDH1A1; ALDH2; AMD1; C3; CBX1; CCKBR; CHRM1
Top DGIdb target count	Doxorubicin hydrochloride	25	0.025	ABCB1; APEX1; ATXN2; BAZ2B; BRAF; CASP3; CPT1A; CYP2D6; EHMT2; FLT3
Selected psychotropic/bipolar-related overlap	Carbamazepine	6	-	CYP2D6; CYP3A5; HLA-A; HLA-B; HSPA1L; TNF
Selected psychotropic/bipolar-related overlap	Lamotrigine	2	-	CYP2D6; HLA-C
Selected psychotropic/bipolar-related overlap	Lithium	11	-	ABCB1; DRD2; FAS; FKBP5; GNRH1; HTR1B; MAPK14; NTRK2; NTS; RABEP1; TPH1
Selected psychotropic/bipolar-related overlap	Olanzapine	23	-	A1BG; A2M; ADRA2B; AMD1; CCKBR; CHRM1; CHRM3; CHRM5; DRD2; DRD5; EIF2AK4; GNB3; HRH1; HTR1B; HTR1D; HTR1E; HTR3A; HTR6; IL1A; PPARG; PRKAA2; RABEP1; SLC1A1

Cross-trait context

Across the broader head-to-head analysis of 11 traits, only four nominally significant NMN-over-AntiDep wins were observed: OCD autophagy, Alzheimer’s disease PPAR signaling, hoarding PPAR signaling, and hoarding autophagy (Table 6). For OCD, the autophagy result was the clearest finding because it was supported by the primary head-to-head test, overlap removal, and the absolute Z-distribution check. In the cross-trait aggregate, KEGG_AUTOPHAGY_ANIMAL and KEGG_PPAR_SIGNALING_PATHWAY each had two nominal wins across 11 traits, with mean dNES values of +0.028 and +0.027, respectively. This broader pattern reinforces the modest nature of the effect. It does not suggest a large pan-psychiatric signal, but it does make the OCD autophagy finding worth following up.

**Table 6:** Cross-trait nominally significant NMN-over-AntiDep_Genes wins and aggregate summary. NMN: Nicotinamide mononucleotide; AntiDep_Genes: Antidepressant-related gene comparator; KEGG: Kyoto Encyclopedia of Genes and Genomes; NES: Normalized enrichment score; dNES: Difference in normalized enrichment score; p1: p-value for set 1; OCD: Obsessive-compulsive disorder; ALZ/alz: Alzheimer’s disease; PPAR: Peroxisome proliferator-activated receptor.

Row type	Trait	NMN pathway set	NES1	p1	dNES	Traits tested	Nominal wins	Mean dNES
Nominally significant win	OCD	KEGG_AUTOPHAGY_ANIMAL	1.166	0.042	0.21	-	-	-
Nominally significant win	Alzheimer’s disease	KEGG_PPAR_SIGNALING_PATHWAY	1.292	0.0285	0.384	-	-	-
Nominally significant win	Hoarding	KEGG_PPAR_SIGNALING_PATHWAY	1.394	0.0025	0.233	-	-	-
Nominally significant win	Hoarding	KEGG_AUTOPHAGY_ANIMAL	1.189	0.0255	0.027	-	-	-
Aggregate	-	KEGG_AUTOPHAGY_ANIMAL	-	-	-	11	2	0.028
Aggregate	-	KEGG_PPAR_SIGNALING_PATHWAY	-	-	-	11	2	0.027

These cross-trait counts were descriptive and were not treated as independent confirmatory evidence or as family-wise or FDR-controlled discoveries.

Detailed supplementary analyses are provided in the appendices. The complete cross-trait head-to-head pathway results are presented in Appendix 1; the nominally significant head-to-head wins and cross-trait aggregate summaries are presented in Appendices 2 and 3; the OCD leading-edge directionality analysis is presented in Appendix 4; the leading-edge overlap analysis in Appendix 5; the overlap-removal robustness analysis in Appendix 6; the absolute Z-score distribution comparisons in Appendix 7; and the comparator leave-one-out analyses in Appendix 8. Additional supplementary results include the principal OCD leading-edge genes (Appendix 9), recurrent leading-edge genes across NMN pathway sets (Appendix 10), functional enrichment results (Appendix 11), OCD tissue-specificity results (Appendix 12), hub-gene tissue profiles (Appendix 13), prioritized genes by maximum absolute Z-score (Appendix 14), DGIdb drug-ranking results (Appendix 15), psychotropic-drug overlap results (Appendix 16), and the signed-ranking re-analysis (Appendix 17).

## Discussion

**Figure 2:**
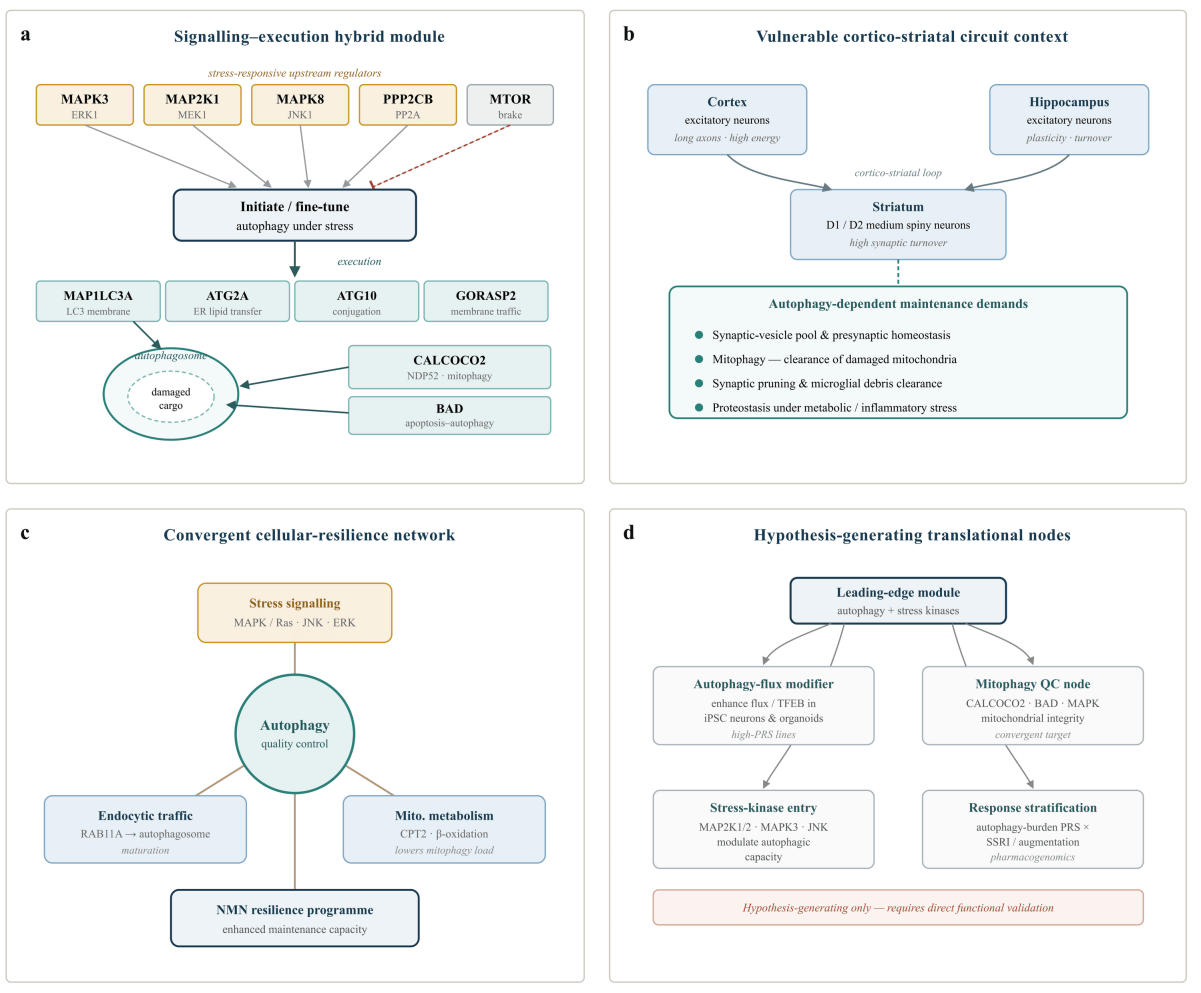
Proposed mechanistic architecture linking an autophagy quality-control program to OCD-relevant neuronal biology. (a) The proposed enrichment reflects a signaling-execution hybrid rather than core machinery alone. Stress-responsive regulators, including MAPK3/ERK1, MAP2K1/MEK1, MAPK8/JNK1, the PP2A subunit PPP2CB, and mTOR, converge on autophagy initiation and fine-tuning. Execution components, including MAP1LC3A, ATG2A, ATG10, GORASP2, CALCOCO2/NDP52, and BAD, support autophagosome formation, mitophagy, and apoptosis-autophagy signaling. (b) The implicated cell types, cortical and hippocampal excitatory neurons and striatal D1/D2 medium spiny neurons, share high synaptic turnover and energy demand. In these neurons, autophagy is hypothesized to support synaptic-vesicle homeostasis, mitophagy, synaptic pruning, and proteostasis under metabolic or inflammatory stress. (c) Autophagy is positioned as a hub in an interconnected resilience network: stress-kinase signaling feeds into it, RAB11A-dependent endocytic trafficking supports autophagosome maturation, and CPT2-driven mitochondrial β-oxidation may reduce mitophagy load. Together, these represent a broader NMN-associated maintenance program whose rate-limiting pathway may differ by tissue and disease context. (d) Four hypothesis-generating entry points follow from this architecture: enhancement of autophagic flux, or TFEB, in patient-derived neurons; targeting mitophagy quality control; modulation of stress kinases; and stratification of treatment response by autophagy-related polygenic burden. These are not treatment recommendations and require direct functional validation. The model shown in this figure is proposed and hypothesis-generating; it is not a validated mechanistic or therapeutic model. OCD; Obsessive-compulsive disorder; NMN: Nicotinamide mononucleotide. Credit: Ngo Cheung. Figure prepared using Microsoft PowerPoint. No AI tools were used.

The main observation from this analysis is simple: in OCD, an NMN-motivated autophagy pathway expansion showed a modest but internally consistent enrichment of TWAS association magnitude relative to antidepressant-related genes. The result persisted after removal of overlapping antidepressant genes, showed minimal leading-edge overlap with the comparator, and was supported by a higher absolute Z-score distribution. No other NMN-motivated pathway showed the same pattern in OCD. The result is therefore best understood as selective autophagy enrichment, not broad enrichment of all NMN-derived pathway biology (Figure 2).

Because the Benjamini-Hochberg-adjusted p-value across the ten OCD pathway tests was 0.41979, these internal checks do not establish statistical significance after multiplicity correction.

Limitations

This study has several important limitations that temper interpretation of the autophagy enrichment signal.

TWAS statistics reflect genetically predicted gene expression rather than directly measured transcript levels in OCD cases and controls. They therefore provide an indirect molecular readout and may be influenced by the properties of the underlying eQTL models.

Gene-level associations, even when aggregated at the pathway level, do not imply causality. Functional validation in OCD-relevant cellular and animal systems is required before the implicated pathways can be assigned a mechanistic role.

KEGG pathways are expert-curated but necessarily incomplete and biased toward better-studied biological processes. Results can therefore be sensitive to pathway boundaries, annotation density, and the precise gene-set definitions used.

The NMN-motivated sets were derived from pathway expansions of only 35 robust genes identified in a single re-analysis of GSE85718 [7]. Many genes within the tested KEGG pathways were not empirically shown to respond to NMN, so the analysis tests pathway themes motivated by NMN biology rather than direct NMN-responsive gene sets.

Cross-tissue meta-Z scores may dilute brain- or cell-type-specific signals. Inferences about cortical, hippocampal, or striatal neurons remain indirect, even though these populations provide a biologically plausible context for the findings [3].

The absolute |Z| ranking framework and competitive GSEA detect association magnitude but cannot distinguish predicted upregulation from downregulation of the pathway in OCD. The signed-ranking analysis likewise did not provide evidence for a uniform directional effect.

No direct functional assays of autophagic flux, mitophagy, lysosomal function, or stress-kinase activity in OCD-relevant models were performed. All conclusions therefore remain correlative and hypothesis-generating.

The primary p-value and Mann-Whitney p-value were close to 0.05, ten pathways were evaluated in parallel, and the autophagy result did not survive FDR correction across those tests. No independent replication cohort or functional validation dataset was analyzed.

The AntiDep_Genes comparator was custom-curated from ATC N06A drug annotations and DGIdb interactions. Different drug lists, interaction filters, or database versions could alter the comparator and therefore the head-to-head result.

These limitations are typical of exploratory TWAS-based pathway analyses but should remain central when interpreting the signal and planning follow-up studies.

The leading-edge composition gives the finding its biological meaning. Autophagy is not a single uniform process, but a family of lysosome-directed degradation pathways that includes macroautophagy, selective autophagy, mitophagy, and related routes for protein and organelle quality control [11]. The strongest autophagy genes in this analysis included MAP1LC3A, ATG2A, ATG10, CALCOCO2, RAB7A, VPS33A, VPS39, CTSD, and other genes involved in autophagosome formation, cargo recognition, vesicle maturation, and lysosomal degradation. Alongside them were MAPK3, MAP2K1, MAPK8, MAP2K2, HRAS, AKT3, PIK3R1, PRKACA, and phosphatase subunits PPP2CB and PPP2CA. This mixture suggests that OCD-related genetic variation may involve the regulation of autophagy as much as its core machinery. In other words, the signal may concern when neurons initiate or fine-tune quality control under stress, rather than simply whether they possess autophagy components.

This interpretation fits with the cell types implicated by Strom NI et al. [3] and with broader genetic and neurobiological models of OCD that emphasize cortico-striato-thalamo-cortical circuitry, glutamatergic and dopaminergic modulation, and distributed network dysfunction rather than a single-transmitter abnormality [12,13]. Cortical and hippocampal excitatory neurons have long projections, high synaptic turnover, and heavy metabolic needs. Striatal D1 and D2 medium spiny neurons sit at the center of cortico-striatal circuitry and integrate dopamine-modulated plasticity with glutamatergic input. These cells must maintain synaptic proteins, recycle vesicle components, clear damaged mitochondria, and manage local stress. Autophagy and endolysosomal trafficking are well suited to this role because they support presynaptic protein turnover, synaptic vesicle homeostasis, axonal cargo transport, and compartment-specific neuronal quality control [4,14,15]. Selective autophagy and mitophagy provide a mechanism for removing dysfunctional mitochondria and damaged cargo in long-lived postmitotic neurons [5,11]. Microglial and neuronal autophagy also contribute to synaptic pruning and circuit refinement, linking cellular clearance to the development and maintenance of neural circuits [6].

The relationship to NMN biology is indirect but still useful. In the Cheung [7] re-analysis of the Mills KF et al. NMN dataset [8], the strongest robust genes were RAB11A and CPT2. RAB11A points toward endosomal recycling and membrane trafficking; CPT2 points toward fatty acid oxidation and mitochondrial substrate use. The present OCD result did not identify endocytosis or fatty acid degradation as the top OCD pathways. Instead, autophagy emerged. This should not be treated as a contradiction. Autophagy, endocytosis, and mitochondrial quality control are closely connected. RAB11A-positive compartments can provide a platform for autophagosome assembly through WIPI2-mediated recognition of PI3P-RAB11A coincidence [9,10]. Mitochondrial fatty acid oxidation may influence mitochondrial stress burden and downstream demand for mitochondrial quality control. MAPK and Ras signaling can regulate stress responses that intersect with autophagy. The OCD result may therefore represent a downstream convergence point of the same cellular resilience themes that appeared in the NMN aging analysis.

Clinically, this matters because treatment resistance in OCD is unlikely to be explained entirely by serotonin transporter biology or dopamine receptor effects. Current treatments act on important systems and remain essential, especially exposure and response prevention, SSRIs, and selected augmentation strategies [1,2]. However, contemporary OCD models increasingly frame the disorder as a circuit-level and neurocognitive condition with interacting genetic, developmental, synaptic, and pharmacological dimensions [12,16]. A patient whose cortico-striatal circuits are genetically burdened by autophagy-regulatory genes might still benefit from SSRIs or exposure therapy, but the biological bottleneck may not be monoaminergic. This could help explain why response is partial or unstable in some individuals. The present analysis cannot prove that claim. It does, however, offer a testable hypothesis: autophagy-related polygenic or transcriptomic burden may define a subgroup of OCD patients whose symptoms are tied more strongly to cellular maintenance capacity.

One translational direction is stratification. Future studies could build autophagy/MAPK pathway burden scores from OCD GWAS or TWAS data and test whether these scores predict symptom dimensions, age at onset, comorbidity, inflammatory history, or treatment response. Existing pharmacogenomic cohorts could be used to ask whether patients with higher autophagy-related burden respond differently to SSRIs, antipsychotic augmentation, glutamatergic augmentation, neuromodulation, or intensive behavioral therapy. Such studies would need careful design because autophagy is broad and context-dependent. A high pathway burden does not automatically mean that increasing autophagy would help. It may mean that the pathway is dysregulated, compensatory, overloaded, or differently engaged across cell types.

A second translational direction is functional modeling. Patient-derived induced pluripotent stem cell (iPSC) neurons, cortico-striatal organoids, or co-culture systems including microglia could be stratified by OCD polygenic risk and autophagy pathway burden. These models could test basal autophagy flux, mitophagy after stress, lysosomal acidification, synaptic vesicle recycling, mitochondrial membrane potential, and responses to inflammatory or metabolic challenge. Autophagy studies should use complementary flux assays rather than relying on the static abundance of single markers such as LC3 alone, because interpretation depends on whether autophagosome formation, cargo delivery, lysosomal fusion, or degradation is being measured [17]. The leading-edge genes suggest specific readouts. MAPK3, MAP2K1, MAPK8, and MAP2K2 point to stress-kinase regulation. MAP1LC3A, ATG2A, ATG10, CALCOCO2, RAB7A, VPS33A, and VPS39 point to autophagosome formation, selective cargo handling, and maturation. BAD and DAPK2 point to the border between stress adaptation and cell-death signaling. These genes can be tested directly rather than left as abstract pathway labels.

A third direction is target discovery, but this must be stated carefully. The current results do not justify treating OCD with NMN, NAD+ precursors, rapamycin-like agents, kinase inhibitors, or any autophagy modulator. Autophagy can be protective or harmful depending on biological context, disease stage, tissue, timing, and dose [18]. In neurons, too little clearance can allow damaged proteins and organelles to accumulate, but excessive or mistimed autophagy could disrupt synapses. The most reasonable therapeutic hypothesis is not “activate autophagy in OCD.” It is that autophagy flux, mitophagy, lysosomal function, and stress-responsive kinase signaling should be measured in OCD-relevant systems, and that future adjunctive strategies may need to normalize rather than simply increase these processes.

The DGIdb results illustrate the same caution. Many top multi-target compounds, including sorafenib, dasatinib, and other kinase or oncology agents, are not plausible psychiatric treatments in their current form. Their appearance in the screen reflects the druggability of kinases and signaling hubs, not clinical readiness. More relevant is the observation that existing psychiatric drugs such as lithium and olanzapine intersect with genes in the leading-edge union. Even here, the interpretation should be modest. The overlap suggests that current treatments may already touch parts of the cellular maintenance network, but it does not show that these effects mediate clinical response.

Despite those caveats, the autophagy result is biologically coherent, although it is not statistically definitive after multiplicity correction. It aligns with neuronal and microglial roles of autophagy in synaptic pruning, presynaptic function, and mitochondrial quality control. It fits the neuronal cell types highlighted by Strom NI et al. [3]. It is independent of most antidepressant-related leading-edge genes. It also connects, mechanistically rather than therapeutically, to NAD+-associated aging biology through trafficking and mitochondrial maintenance pathways. That makes it a useful bridge between psychiatric genetics and cellular resilience biology.

## Conclusions

In summary, this analysis identifies autophagy-related transcriptional programs as a modest but internally consistent signal within OCD TWAS data when compared with an antidepressant-related gene set. The enrichment survived removal of overlapping antidepressant genes and was supported by a higher absolute Z-score distribution, with leading-edge genes implicating both upstream stress-responsive signaling, including MAPK-family and PP2A-related nodes, and core autophagy execution components. However, the primary p-value did not survive Benjamini-Hochberg FDR correction across the ten tested pathways, and the finding requires replication in independent datasets and functional systems. The results support further investigation of neuronal quality-control pathways, including autophagy, mitophagy, and lysosomal biology, as contributors to OCD genetic architecture rather than as immediate therapeutic targets. No evidence is provided that NMN, NAD+ precursors, or pharmacological autophagy modulators would be beneficial in OCD.

## Appendices

Appendix 1

**Table 7:** Exhaustive head-to-head pathway results across traits. Note. Bold rows indicate nominal pathway wins under the specified criterion: NES1 greater than NES2 and p1 less than 0.05. NMN: Nicotinamide mononucleotide; KEGG: Kyoto Encyclopedia of Genes and Genomes; NES: Normalized enrichment score; dNES: Difference in normalized enrichment score; MDD: Major depressive disorder; BIP: Bipolar disorder; SCZ: Schizophrenia; OCD: Obsessive-compulsive disorder; PPD: Postpartum depression; PTSD: Post-traumatic stress disorder; ALZ: Alzheimer’s disease; AN: Anorexia nervosa.

Trait	NMN pathway set	NES1	p1	NES2	p2	dNES	Verdict
mdd	KEGG_FATTY_ACID_DEGRADATION	1.1143	0.245377	1.0098	0.455772	0.1045	not_better
mdd	KEGG_ENDOCYTOSIS	1.0614	0.230885	1.0098	0.455772	0.0516	not_better
mdd	KEGG_RAS_SIGNALING_PATHWAY	1.0329	0.355322	1.0098	0.455772	0.0231	not_better
mdd	KEGG_PPAR_SIGNALING_PATHWAY	0.9865	0.536732	1.0098	0.455772	-0.0233	not_better
mdd	KEGG_NEUROACTIVE_LIGAND_RECEPTOR_INTERACTION	0.9423	0.775112	1.0098	0.455772	-0.0675	not_better
mdd	KEGG_MAPK_SIGNALING_PATHWAY	0.9129	0.861069	1.0098	0.455772	-0.0969	not_better
mdd	KEGG_CITRATE_CYCLE_TCA_CYCLE	0.8960	0.699150	1.0098	0.455772	-0.1138	not_better
mdd	KEGG_REGULATION_OF_ACTIN_CYTOSKELETON	0.8913	0.882059	1.0098	0.455772	-0.1185	not_better
mdd	KEGG_SYNAPTIC_VESICLE_CYCLE	0.8819	0.785107	1.0098	0.455772	-0.1279	not_better
mdd	KEGG_AUTOPHAGY_ANIMAL	0.8469	0.939030	1.0098	0.455772	-0.1629	not_better
bip	KEGG_PPAR_SIGNALING_PATHWAY	1.1889	0.101949	0.9454	0.713143	0.2435	not_better
bip	KEGG_AUTOPHAGY_ANIMAL	1.1101	0.131934	0.9454	0.713143	0.1647	not_better
bip	KEGG_FATTY_ACID_DEGRADATION	1.1045	0.286357	0.9454	0.713143	0.1591	not_better
bip	KEGG_MAPK_SIGNALING_PATHWAY	1.0600	0.236382	0.9454	0.713143	0.1146	not_better
bip	KEGG_REGULATION_OF_ACTIN_CYTOSKELETON	1.0367	0.343328	0.9454	0.713143	0.0913	not_better
bip	KEGG_ENDOCYTOSIS	1.0263	0.377811	0.9454	0.713143	0.0809	not_better
bip	KEGG_RAS_SIGNALING_PATHWAY	1.0211	0.406297	0.9454	0.713143	0.0757	not_better
bip	KEGG_CITRATE_CYCLE_TCA_CYCLE	1.0036	0.498751	0.9454	0.713143	0.0582	not_better
bip	KEGG_SYNAPTIC_VESICLE_CYCLE	0.9935	0.522739	0.9454	0.713143	0.0481	not_better
bip	KEGG_NEUROACTIVE_LIGAND_RECEPTOR_INTERACTION	0.8310	0.987506	0.9454	0.713143	-0.1144	not_better
scz	KEGG_CITRATE_CYCLE_TCA_CYCLE	1.1008	0.310345	1.0919	0.162919	0.0089	not_better
scz	KEGG_FATTY_ACID_DEGRADATION	1.0644	0.370815	1.0919	0.162919	-0.0275	not_better
scz	KEGG_ENDOCYTOSIS	1.0120	0.447276	1.0919	0.162919	-0.0799	not_better
scz	KEGG_AUTOPHAGY_ANIMAL	1.0091	0.471264	1.0919	0.162919	-0.0828	not_better
scz	KEGG_MAPK_SIGNALING_PATHWAY	0.9632	0.678161	1.0919	0.162919	-0.1287	not_better
scz	KEGG_RAS_SIGNALING_PATHWAY	0.9377	0.748626	1.0919	0.162919	-0.1542	not_better
scz	KEGG_SYNAPTIC_VESICLE_CYCLE	0.9057	0.741629	1.0919	0.162919	-0.1862	not_better
scz	KEGG_REGULATION_OF_ACTIN_CYTOSKELETON	0.8825	0.903548	1.0919	0.162919	-0.2094	not_better
scz	KEGG_NEUROACTIVE_LIGAND_RECEPTOR_INTERACTION	0.8714	0.948026	1.0919	0.162919	-0.2205	not_better
scz	KEGG_PPAR_SIGNALING_PATHWAY	0.7299	0.976512	1.0919	0.162919	-0.3620	not_better
ocd	KEGG_AUTOPHAGY_ANIMAL	1.1661	0.041979	0.9564	0.677661	0.2097	set1_beats_set2
ocd	KEGG_RAS_SIGNALING_PATHWAY	1.1167	0.100950	0.9564	0.677661	0.1603	not_better
ocd	KEGG_MAPK_SIGNALING_PATHWAY	1.0893	0.151924	0.9564	0.677661	0.1329	not_better
ocd	KEGG_ENDOCYTOSIS	1.0197	0.399800	0.9564	0.677661	0.0633	not_better
ocd	KEGG_SYNAPTIC_VESICLE_CYCLE	0.9505	0.627686	0.9564	0.677661	-0.0059	not_better
ocd	KEGG_FATTY_ACID_DEGRADATION	0.9282	0.660170	0.9564	0.677661	-0.0282	not_better
ocd	KEGG_REGULATION_OF_ACTIN_CYTOSKELETON	0.9014	0.870065	0.9564	0.677661	-0.0550	not_better
ocd	KEGG_NEUROACTIVE_LIGAND_RECEPTOR_INTERACTION	0.8944	0.897551	0.9564	0.677661	-0.0620	not_better
ocd	KEGG_CITRATE_CYCLE_TCA_CYCLE	0.8236	0.815092	0.9564	0.677661	-0.1328	not_better
ocd	KEGG_PPAR_SIGNALING_PATHWAY	0.7535	0.957021	0.9564	0.677661	-0.2029	not_better
ppd	KEGG_RAS_SIGNALING_PATHWAY	1.0642	0.222889	0.8767	0.917541	0.1875	not_better
ppd	KEGG_ENDOCYTOSIS	0.9984	0.505247	0.8767	0.917541	0.1217	not_better
ppd	KEGG_MAPK_SIGNALING_PATHWAY	0.9889	0.546227	0.8767	0.917541	0.1122	not_better
ppd	KEGG_CITRATE_CYCLE_TCA_CYCLE	0.9727	0.551224	0.8767	0.917541	0.0960	not_better
ppd	KEGG_AUTOPHAGY_ANIMAL	0.9512	0.686157	0.8767	0.917541	0.0745	not_better
ppd	KEGG_NEUROACTIVE_LIGAND_RECEPTOR_INTERACTION	0.9504	0.743128	0.8767	0.917541	0.0737	not_better
ppd	KEGG_SYNAPTIC_VESICLE_CYCLE	0.9390	0.674663	0.8767	0.917541	0.0623	not_better
ppd	KEGG_REGULATION_OF_ACTIN_CYTOSKELETON	0.9360	0.769615	0.8767	0.917541	0.0593	not_better
ppd	KEGG_FATTY_ACID_DEGRADATION	0.9078	0.698651	0.8767	0.917541	0.0311	not_better
ppd	KEGG_PPAR_SIGNALING_PATHWAY	0.8100	0.914043	0.8767	0.917541	-0.0667	not_better
ptsd	KEGG_CITRATE_CYCLE_TCA_CYCLE	1.2447	0.109945	1.0384	0.339330	0.2063	not_better
ptsd	KEGG_ENDOCYTOSIS	1.0967	0.128436	1.0384	0.339330	0.0583	not_better
ptsd	KEGG_PPAR_SIGNALING_PATHWAY	1.0912	0.252874	1.0384	0.339330	0.0528	not_better
ptsd	KEGG_SYNAPTIC_VESICLE_CYCLE	1.0013	0.497251	1.0384	0.339330	-0.0371	not_better
ptsd	KEGG_RAS_SIGNALING_PATHWAY	0.9603	0.661169	1.0384	0.339330	-0.0781	not_better
ptsd	KEGG_MAPK_SIGNALING_PATHWAY	0.9191	0.832084	1.0384	0.339330	-0.1193	not_better
ptsd	KEGG_NEUROACTIVE_LIGAND_RECEPTOR_INTERACTION	0.8992	0.890055	1.0384	0.339330	-0.1392	not_better
ptsd	KEGG_REGULATION_OF_ACTIN_CYTOSKELETON	0.8680	0.924538	1.0384	0.339330	-0.1704	not_better
ptsd	KEGG_AUTOPHAGY_ANIMAL	0.8471	0.945527	1.0384	0.339330	-0.1913	not_better
ptsd	KEGG_FATTY_ACID_DEGRADATION	0.8149	0.843578	1.0384	0.339330	-0.2235	not_better
alz	KEGG_PPAR_SIGNALING_PATHWAY	1.2915	0.028486	0.9075	0.837081	0.3840	set1_beats_set2
alz	KEGG_ENDOCYTOSIS	1.0921	0.137931	0.9075	0.837081	0.1846	not_better
alz	KEGG_CITRATE_CYCLE_TCA_CYCLE	1.0800	0.345827	0.9075	0.837081	0.1725	not_better
alz	KEGG_REGULATION_OF_ACTIN_CYTOSKELETON	1.0446	0.300850	0.9075	0.837081	0.1371	not_better
alz	KEGG_AUTOPHAGY_ANIMAL	1.0417	0.349825	0.9075	0.837081	0.1342	not_better
alz	KEGG_RAS_SIGNALING_PATHWAY	1.0185	0.421289	0.9075	0.837081	0.1110	not_better
alz	KEGG_FATTY_ACID_DEGRADATION	0.9078	0.695152	0.9075	0.837081	0.0003	not_better
alz	KEGG_MAPK_SIGNALING_PATHWAY	0.8991	0.899550	0.9075	0.837081	-0.0084	not_better
alz	KEGG_SYNAPTIC_VESICLE_CYCLE	0.8512	0.839080	0.9075	0.837081	-0.0563	not_better
alz	KEGG_NEUROACTIVE_LIGAND_RECEPTOR_INTERACTION	0.7652	0.998001	0.9075	0.837081	-0.1423	not_better
an	KEGG_SYNAPTIC_VESICLE_CYCLE	1.1296	0.175912	1.0886	0.173413	0.0410	not_better
an	KEGG_AUTOPHAGY_ANIMAL	1.0079	0.479760	1.0886	0.173413	-0.0807	not_better
an	KEGG_ENDOCYTOSIS	1.0002	0.503248	1.0886	0.173413	-0.0884	not_better
an	KEGG_PPAR_SIGNALING_PATHWAY	0.9806	0.548226	1.0886	0.173413	-0.1080	not_better
an	KEGG_MAPK_SIGNALING_PATHWAY	0.9728	0.639180	1.0886	0.173413	-0.1158	not_better
an	KEGG_RAS_SIGNALING_PATHWAY	0.9365	0.747126	1.0886	0.173413	-0.1521	not_better
an	KEGG_REGULATION_OF_ACTIN_CYTOSKELETON	0.8997	0.861069	1.0886	0.173413	-0.1889	not_better
an	KEGG_FATTY_ACID_DEGRADATION	0.8918	0.736632	1.0886	0.173413	-0.1968	not_better
an	KEGG_NEUROACTIVE_LIGAND_RECEPTOR_INTERACTION	0.8396	0.979010	1.0886	0.173413	-0.2490	not_better
an	KEGG_CITRATE_CYCLE_TCA_CYCLE	0.6885	0.941529	1.0886	0.173413	-0.4001	not_better
hoarding	KEGG_PPAR_SIGNALING_PATHWAY	1.3937	0.002499	1.1612	0.048476	0.2325	set1_beats_set2
hoarding	KEGG_FATTY_ACID_DEGRADATION	1.2108	0.115442	1.1612	0.048476	0.0496	not_better
hoarding	KEGG_AUTOPHAGY_ANIMAL	1.1886	0.025487	1.1612	0.048476	0.0274	set1_beats_set2
hoarding	KEGG_SYNAPTIC_VESICLE_CYCLE	1.0792	0.290855	1.1612	0.048476	-0.0820	not_better
hoarding	KEGG_NEUROACTIVE_LIGAND_RECEPTOR_INTERACTION	1.0287	0.353323	1.1612	0.048476	-0.1325	not_better
hoarding	KEGG_REGULATION_OF_ACTIN_CYTOSKELETON	1.0108	0.451274	1.1612	0.048476	-0.1504	not_better
hoarding	KEGG_ENDOCYTOSIS	0.9711	0.623688	1.1612	0.048476	-0.1901	not_better
hoarding	KEGG_RAS_SIGNALING_PATHWAY	0.9417	0.731134	1.1612	0.048476	-0.2195	not_better
hoarding	KEGG_MAPK_SIGNALING_PATHWAY	0.8904	0.907546	1.1612	0.048476	-0.2708	not_better
hoarding	KEGG_CITRATE_CYCLE_TCA_CYCLE	0.8758	0.743128	1.1612	0.048476	-0.2854	not_better
tics	KEGG_FATTY_ACID_DEGRADATION	1.1653	0.193403	0.8493	0.945027	0.3160	not_better
tics	KEGG_ENDOCYTOSIS	1.0572	0.257871	0.8493	0.945027	0.2079	not_better
tics	KEGG_AUTOPHAGY_ANIMAL	1.0316	0.381309	0.8493	0.945027	0.1823	not_better
tics	KEGG_CITRATE_CYCLE_TCA_CYCLE	1.0215	0.457271	0.8493	0.945027	0.1722	not_better
tics	KEGG_PPAR_SIGNALING_PATHWAY	0.9965	0.516242	0.8493	0.945027	0.1472	not_better
tics	KEGG_RAS_SIGNALING_PATHWAY	0.9783	0.584208	0.8493	0.945027	0.1290	not_better
tics	KEGG_SYNAPTIC_VESICLE_CYCLE	0.9290	0.686657	0.8493	0.945027	0.0797	not_better
tics	KEGG_REGULATION_OF_ACTIN_CYTOSKELETON	0.8583	0.925537	0.8493	0.945027	0.0090	not_better
tics	KEGG_NEUROACTIVE_LIGAND_RECEPTOR_INTERACTION	0.8449	0.980010	0.8493	0.945027	-0.0044	not_better
tics	KEGG_MAPK_SIGNALING_PATHWAY	0.7180	1.000000	0.8493	0.945027	-0.1313	not_better
anx_cc	KEGG_CITRATE_CYCLE_TCA_CYCLE	1.0795	0.339830	0.9683	0.637181	0.1112	not_better
anx_cc	KEGG_FATTY_ACID_DEGRADATION	1.0497	0.388806	0.9683	0.637181	0.0814	not_better
anx_cc	KEGG_RAS_SIGNALING_PATHWAY	1.0162	0.437281	0.9683	0.637181	0.0479	not_better
anx_cc	KEGG_AUTOPHAGY_ANIMAL	0.9990	0.511744	0.9683	0.637181	0.0307	not_better
anx_cc	KEGG_PPAR_SIGNALING_PATHWAY	0.9707	0.576212	0.9683	0.637181	0.0024	not_better
anx_cc	KEGG_REGULATION_OF_ACTIN_CYTOSKELETON	0.9507	0.704148	0.9683	0.637181	-0.0176	not_better
anx_cc	KEGG_ENDOCYTOSIS	0.9465	0.733133	0.9683	0.637181	-0.0218	not_better
anx_cc	KEGG_MAPK_SIGNALING_PATHWAY	0.8669	0.953523	0.9683	0.637181	-0.1014	not_better
anx_cc	KEGG_SYNAPTIC_VESICLE_CYCLE	0.8655	0.818091	0.9683	0.637181	-0.1028	not_better
anx_cc	KEGG_NEUROACTIVE_LIGAND_RECEPTOR_INTERACTION	0.8399	0.979510	0.9683	0.637181	-0.1284	not_better

Appendix 2 

**Table 8:** Nominally significant head-to-head wins. NMN: Nicotinamide mononucleotide; KEGG: Kyoto Encyclopedia of Genes and Genomes; NES: Normalized enrichment score; p1: p-value for set 1; dNES: Difference in normalized enrichment score; ocd: Obsessive-compulsive disorder; alz: Alzheimer’s disease; PPAR: Peroxisome proliferator-activated receptor; AntiDep_Genes: Antidepressant-related gene comparator.

Trait	NMN pathway set	Comparator	NES1	p1	dNES
ocd	KEGG_AUTOPHAGY_ANIMAL	AntiDep_Genes	1.166	0.0420	0.210
alz	KEGG_PPAR_SIGNALING_PATHWAY	AntiDep_Genes	1.292	0.0285	0.384
hoarding	KEGG_PPAR_SIGNALING_PATHWAY	AntiDep_Genes	1.394	0.0025	0.233
hoarding	KEGG_AUTOPHAGY_ANIMAL	AntiDep_Genes	1.189	0.0255	0.027

Appendix 3  

**Table 9:** Cross-trait aggregate summary. NMN: Nicotinamide mononucleotide; KEGG: Kyoto Encyclopedia of Genes and Genomes; AntiDep_Genes: Antidepressant-related gene comparator; dNES: Difference in normalized enrichment score; PPAR: Peroxisome proliferator-activated receptor; TCA: Tricarboxylic acid; MAPK: Mitogen-activated protein kinase.

NMN pathway set	Comparator	Traits tested	Nominal wins	Mean dNES
KEGG_AUTOPHAGY_ANIMAL	AntiDep_Genes	11	2	0.028
KEGG_PPAR_SIGNALING_PATHWAY	AntiDep_Genes	11	2	0.027
KEGG_ENDOCYTOSIS	AntiDep_Genes	11	0	0.035
KEGG_FATTY_ACID_DEGRADATION	AntiDep_Genes	11	0	0.024
KEGG_RAS_SIGNALING_PATHWAY	AntiDep_Genes	11	0	0.012
KEGG_CITRATE_CYCLE_TCA_CYCLE	AntiDep_Genes	11	0	-0.01
KEGG_SYNAPTIC_VESICLE_CYCLE	AntiDep_Genes	11	0	-0.033
KEGG_MAPK_SIGNALING_PATHWAY	AntiDep_Genes	11	0	-0.056
KEGG_REGULATION_OF_ACTIN_CYTOSKELETON	AntiDep_Genes	11	0	-0.056
KEGG_NEUROACTIVE_LIGAND_RECEPTOR_INTERACTION	AntiDep_Genes	11	0	-0.108

Appendix 4 

**Table 10:** OCD leading-edge directionality using signed meta-Z values. Note. No gene set showed a significant positive or negative sign imbalance among the leading-edge genes. NMN: Nicotinamide mononucleotide; KEGG: Kyoto Encyclopedia of Genes and Genomes; AntiDep_Genes: Antidepressant-related gene comparator; MAPK: Mitogen-activated protein kinase; PPAR: Peroxisome proliferator-activated receptor; TCA: Tricarboxylic acid; Z: Z-score; p: p-value; OCD: Obsessive-compulsive disorder.

Group	Gene set	Number of leading-edge genes	Number positive	Number negative	Percentage negative	Mean Z	Sign-test p-value
NMN	KEGG_ENDOCYTOSIS	85	47	38	44.7	0.174	0.38567
NMN	KEGG_FATTY_ACID_DEGRADATION	7	4	3	42.9	0.2709	1
NMN	KEGG_MAPK_SIGNALING_PATHWAY	73	35	38	52.1	-0.0654	0.81512
NMN	KEGG_RAS_SIGNALING_PATHWAY	58	29	29	50	0.0793	1
NMN	KEGG_PPAR_SIGNALING_PATHWAY	29	15	14	48.3	-0.1206	1
NMN	KEGG_NEUROACTIVE_LIGAND_RECEPTOR_INTERACTION	116	62	54	46.6	0.0619	0.51591
NMN	KEGG_REGULATION_OF_ACTIN_CYTOSKELETON	74	32	42	56.8	-0.4088	0.29541
NMN	KEGG_CITRATE_CYCLE_TCA_CYCLE	5	3	2	40	1.2667	1
NMN	KEGG_AUTOPHAGY_ANIMAL	45	23	22	48.9	0.2199	1
NMN	KEGG_SYNAPTIC_VESICLE_CYCLE	27	14	13	48.1	0.2151	1
AntiDep	AntiDep_Genes	78	33	45	57.7	-0.2916	0.21268

Appendix 5 

**Table 11:** OCD leading-edge overlap between NMN pathway sets and AntiDep_Genes. OCD: Obsessive-compulsive disorder; NMN: Nicotinamide mononucleotide; AntiDep_Genes: Antidepressant-related gene comparator; KEGG: Kyoto Encyclopedia of Genes and Genomes; MAPK: Mitogen-activated protein kinase; PPAR: Peroxisome proliferator-activated receptor; TCA: Tricarboxylic acid; MTOR: Mechanistic target of rapamycin.

NMN pathway set	Number of NMN leading-edge genes	Number of AntiDep leading-edge genes	Overlap	Jaccard index	Shared leading-edge genes
KEGG_ENDOCYTOSIS	85	78	3	0.0187	GRK5; HLA-A; RABEP1
KEGG_FATTY_ACID_DEGRADATION	7	78	0	0	None
KEGG_MAPK_SIGNALING_PATHWAY	73	78	2	0.0134	PPM1A; TP53
KEGG_RAS_SIGNALING_PATHWAY	58	78	2	0.0149	ABL2; GNB3
KEGG_PPAR_SIGNALING_PATHWAY	29	78	1	0.0094	PPARD
KEGG_NEUROACTIVE_LIGAND_RECEPTOR_INTERACTION	116	78	18	0.1023	ADRA2B; ADRB2; CHRM1; CHRM3; DRD2; DRD5; GABRA1; GABRA2; GABRA4; GALR1; GRIN1; GRIN2A; GRIN2D; GRIN3A; HRH1; HTR1B; MC1R; MTNR1B
KEGG_REGULATION_OF_ACTIN_CYTOSKELETON	74	78	1	0.0066	CHRM3
KEGG_CITRATE_CYCLE_TCA_CYCLE	5	78	0	0	None
KEGG_AUTOPHAGY_ANIMAL	45	78	1	0.0082	MTOR
KEGG_SYNAPTIC_VESICLE_CYCLE	27	78	0	0	None

Appendix 6  

**Table 12:** OCD robustness analysis after removing AntiDep-overlapping genes. OCD: Obsessive-compulsive disorder; AntiDep: Antidepressant-related gene comparator; NMN: Nicotinamide mononucleotide; KEGG: Kyoto Encyclopedia of Genes and Genomes; n: Number; NES: Normalized enrichment score; p: p-value; dNES: Difference in normalized enrichment score; MAPK: Mitogen-activated protein kinase; PPAR: Peroxisome proliferator-activated receptor; TCA: Tricarboxylic acid.

NMN pathway set	Full n	Removed n	NES full	p full	NES cleaned	p cleaned	dNES after removal	Comparator NES
KEGG_ENDOCYTOSIS	250	6	1.0185	0.428286	1.0043	0.48026	-0.0142	0.9591
KEGG_FATTY_ACID_DEGRADATION	43	1	0.9344	0.643178	0.9344	0.643178	0	0.9591
KEGG_MAPK_SIGNALING_PATHWAY	300	14	1.0903	0.150425	1.0893	0.146427	-0.001	0.9591
KEGG_RAS_SIGNALING_PATHWAY	238	13	1.1175	0.108946	1.1406	0.062969	0.0231	0.9591
KEGG_PPAR_SIGNALING_PATHWAY	76	2	0.7549	0.957521	0.7405	0.961519	-0.0144	0.9591
KEGG_NEUROACTIVE_LIGAND_RECEPTOR_INTERACTION	371	67	0.8931	0.911044	0.9024	0.862069	0.0093	0.9591
KEGG_REGULATION_OF_ACTIN_CYTOSKELETON	232	8	0.9	0.869565	0.9382	0.732134	0.0382	0.9591
KEGG_CITRATE_CYCLE_TCA_CYCLE	30	1	0.8301	0.798101	0.9067	0.662669	0.0766	0.9591
KEGG_AUTOPHAGY_ANIMAL	169	4	1.1665	0.053473	1.1784	0.042479	0.0119	0.9591
KEGG_SYNAPTIC_VESICLE_CYCLE	79	4	0.9522	0.627186	0.949	0.627186	-0.0032	0.9591

Appendix 7  

**Table 13:** OCD absolute Z-score distribution comparison between NMN pathway sets and AntiDep_Genes. OCD: Obsessive-compulsive disorder; NMN: Nicotinamide mononucleotide; AntiDep_Genes: Antidepressant-related gene comparator; KEGG: Kyoto Encyclopedia of Genes and Genomes; MAPK: Mitogen-activated protein kinase; PPAR: Peroxisome proliferator-activated receptor; TCA: Tricarboxylic acid; U: Mann-Whitney U statistic.

NMN pathway set	Median absolute Z, NMN set	Median absolute Z, AntiDep_Genes	Mann-Whitney U p-value
KEGG_ENDOCYTOSIS	1.1227	1.1568	0.73091
KEGG_FATTY_ACID_DEGRADATION	1.0323	1.1568	0.89101
KEGG_MAPK_SIGNALING_PATHWAY	1.0819	1.1568	0.49321
KEGG_RAS_SIGNALING_PATHWAY	1.2019	1.1568	0.21815
KEGG_PPAR_SIGNALING_PATHWAY	1.1115	1.1568	0.96434
KEGG_NEUROACTIVE_LIGAND_RECEPTOR_INTERACTION	1.0879	1.1568	0.64553
KEGG_REGULATION_OF_ACTIN_CYTOSKELETON	1.1213	1.1568	0.47191
KEGG_CITRATE_CYCLE_TCA_CYCLE	0.8919	1.1568	0.63485
KEGG_AUTOPHAGY_ANIMAL	1.3478	1.1568	0.04717
KEGG_SYNAPTIC_VESICLE_CYCLE	1.1383	1.1568	0.63169

Appendix 8  

**Table 14:** AntiDep_Genes leave-one-out NES drivers in OCD. AntiDep_Genes: Antidepressant-related gene comparator; NES: Normalized enrichment score; OCD: Obsessive-compulsive disorder; Z: Z-score.

Removed gene	Z	NES base	NES without gene	Delta NES
ANK3	9.1227	0.9591	0.9349	-0.0242
METTL21A	-5.2441	0.9591	0.9436	-0.0155
RABEP1	-4.7088	0.9591	0.9448	-0.0143
KCNH2	4.6065	0.9591	0.945	-0.0141
GMNN	4.1192	0.9591	0.9461	-0.013
CBX1	4.0136	0.9591	0.9463	-0.0128
GNB3	-3.9512	0.9591	0.9465	-0.0126
EHMT2	3.8034	0.9591	0.9468	-0.0123
MPHOSPH8	3.5399	0.9591	0.9474	-0.0117
MC1R	-3.5319	0.9591	0.9474	-0.0117
CHRM3	3.5311	0.9591	0.9474	-0.0117
DTNBP1	-3.5045	0.9591	0.9474	-0.0117
PPM1A	-3.3692	0.9591	0.9477	-0.0114
ERCC8	3.2908	0.9591	0.9479	-0.0112
SLC39A14	-3.2241	0.9591	0.948	-0.0111
C1QBP	-3.0311	0.9591	0.9485	-0.0106
ATP4A	3.0118	0.9591	0.9485	-0.0106
GSK3B	-2.9303	0.9591	0.9487	-0.0104
CYP2D6	-2.89	0.9591	0.9488	-0.0103
ACE	-2.8832	0.9591	0.9488	-0.0103
ANO2	-2.837	0.9591	0.949	-0.01
MTOR	-2.814	0.9591	0.949	-0.01
UST	2.802	0.9591	0.949	-0.01
HTR1B	2.788	0.9591	0.949	-0.01
MIEF2	-2.651	0.9591	0.949	-0.01

Appendix 9  

**Table 15:** OCD leading-edge genes for the principal positive pathway and comparator. OCD: Obsessive-compulsive disorder; AntiDep/AntiDep_Genes: Antidepressant-related gene comparator; NMN: Nicotinamide mononucleotide; KEGG: Kyoto Encyclopedia of Genes and Genomes; Z: Z-score; MAPK: Mitogen-activated protein kinase; ATG: Autophagy-related; RAB: Ras-related protein.

Group	Gene set	Gene	Z	Absolute Z	Rank
AntiDep	AntiDep_Genes	ANK3	9.123	9.123	12
AntiDep	AntiDep_Genes	METTL21A	-5.244	5.244	320
AntiDep	AntiDep_Genes	RABEP1	-4.709	4.709	491
AntiDep	AntiDep_Genes	KCNH2	4.606	4.606	531
AntiDep	AntiDep_Genes	GMNN	4.119	4.119	770
AntiDep	AntiDep_Genes	CBX1	4.014	4.014	863
AntiDep	AntiDep_Genes	GNB3	-3.951	3.951	921
AntiDep	AntiDep_Genes	EHMT2	3.803	3.803	1053
AntiDep	AntiDep_Genes	MPHOSPH8	3.54	3.54	1271
AntiDep	AntiDep_Genes	MC1R	-3.532	3.532	1281
AntiDep	AntiDep_Genes	CHRM3	3.531	3.531	1283
AntiDep	AntiDep_Genes	DTNBP1	-3.505	3.505	1315
AntiDep	AntiDep_Genes	PPM1A	-3.369	3.369	1469
AntiDep	AntiDep_Genes	ERCC8	3.291	3.291	1554
AntiDep	AntiDep_Genes	SLC39A14	-3.224	3.224	1635
NMN	KEGG_AUTOPHAGY_ANIMAL	MAPK3	6.064	6.064	153
NMN	KEGG_AUTOPHAGY_ANIMAL	MAP1LC3A	5.96	5.96	167
NMN	KEGG_AUTOPHAGY_ANIMAL	PPP2CB	-5.938	5.938	173
NMN	KEGG_AUTOPHAGY_ANIMAL	MAP2K1	-5.646	5.646	220
NMN	KEGG_AUTOPHAGY_ANIMAL	GORASP2	5.506	5.506	247
NMN	KEGG_AUTOPHAGY_ANIMAL	BAD	5.442	5.442	265
NMN	KEGG_AUTOPHAGY_ANIMAL	ATG2A	5.293	5.293	297
NMN	KEGG_AUTOPHAGY_ANIMAL	ATG10	-5.236	5.236	324
NMN	KEGG_AUTOPHAGY_ANIMAL	MAPK8	4.695	4.695	496
NMN	KEGG_AUTOPHAGY_ANIMAL	CALCOCO2	4.578	4.578	545

Appendix 10  

**Table 16:** Recurrent leading-edge genes across OCD NMN sets. OCD: Obsessive-compulsive disorder; NMN: Nicotinamide mononucleotide; AntiDep: Antidepressant-related gene comparator; Z: Z-score; MAPK: Mitogen-activated protein kinase; FGFR: Fibroblast growth factor receptor; EGF: Epidermal growth factor; EGFR: Epidermal growth factor receptor; RAF1: Rapidly accelerated fibrosarcoma 1.

Gene	Number of traits	Number of sets	Mean absolute Z	Maximum absolute Z	Mean Z	Groups
HRAS	1	5	4.026	4.026	-4.026	NMN
MAPK3	1	4	6.064	6.064	6.064	NMN
MAP2K1	1	4	5.646	5.646	-5.646	NMN
MAP2K2	1	4	4.402	4.402	-4.402	NMN
FGFR4	1	4	3.986	3.986	3.986	NMN
MAPK1	1	4	3.3	3.3	3.3	NMN
AKT3	1	4	3.208	3.208	3.208	NMN
RRAS2	1	4	2.522	2.522	-2.522	NMN
RRAS	1	4	2.297	2.297	2.297	NMN
EGFR	1	4	2.116	2.116	2.116	NMN
RAF1	1	4	2.093	2.093	-2.093	NMN
MAPK8	1	3	4.695	4.695	4.695	NMN
FGF8	1	3	3.796	3.796	-3.796	NMN
SOS2	1	3	3.703	3.703	-3.703	NMN
PRKACA	1	3	3.553	3.553	3.553	NMN
CHRM3	1	3	3.531	3.531	3.531	AntiDep; NMN
PRKACB	1	3	3.148	3.148	3.148	NMN
PIK3R1	1	3	3.091	3.091	-3.091	NMN
PDGFRB	1	3	2.531	2.531	-2.531	NMN
EGF	1	3	2.501	2.501	2.501	NMN
PIK3R3	1	3	2.158	2.158	-2.158	NMN
RHOA	1	3	2.001	2.001	-2.001	NMN
FGFR1	1	3	1.923	1.923	1.923	NMN
BAD	1	2	5.442	5.442	5.442	NMN
RABEP1	1	2	4.709	4.709	-4.709	AntiDep; NMN

Appendix 11  

**Table 17:** Functional enrichment of the 795-gene leading-edge union: top terms. KEGG: Kyoto Encyclopedia of Genes and Genomes; MAPK: Mitogen-activated protein kinase; PPAR: Peroxisome proliferator-activated receptor; GPCR: G protein-coupled receptor; R-HSA: Reactome Homo sapiens identifier; GO: Gene Ontology; cAMP: Cyclic adenosine monophosphate.

Library	Term	Adjusted p-value	Overlap
Reactome_2022	Signal Transduction R-HSA-162582	3.18E-119	358/2465
KEGG_2021_Human	Endocytosis	2.20E-109	128/252
KEGG_2021_Human	Regulation of actin cytoskeleton	1.11E-106	119/218
KEGG_2021_Human	MAPK signaling pathway	6.36E-99	128/294
KEGG_2021_Human	Neuroactive ligand-receptor interaction	1.91E-95	133/341
KEGG_2021_Human	Ras signaling pathway	2.15E-88	109/232
KEGG_2021_Human	Autophagy	1.55E-82	85/137
KEGG_2021_Human	PPAR signaling pathway	1.14E-67	59/74
KEGG_2021_Human	Synaptic vesicle cycle	2.43E-63	58/78
Reactome_2022	GPCR Downstream Signaling R-HSA-388396	2.57E-62	138/619
Reactome_2022	Signaling by GPCR R-HSA-372790	8.91E-60	142/689
Reactome_2022	Signaling by Receptor Tyrosine Kinases	3.92E-57	119/496
KEGG_2021_Human	Human cytomegalovirus infection	1.69E-56	83/225
KEGG_2021_Human	Pathways in cancer	6.22E-53	117/531
KEGG_2021_Human	Shigellosis	1.05E-50	81/246
KEGG_2021_Human	Yersinia infection	5.85E-50	63/137
KEGG_2021_Human	Rap1 signaling pathway	6.90E-50	75/210
KEGG_2021_Human	Focal adhesion	3.62E-49	73/201
KEGG_2021_Human	cAMP signaling pathway	6.45E-49	75/216
KEGG_2021_Human	Human immunodeficiency virus 1 infection	2.54E-47	73/212
Reactome_2022	GPCR Ligand Binding R-HSA-500792	3.86E-47	104/458
KEGG_2021_Human	PI3K-Akt signaling pathway	5.43E-47	91/354
Reactome_2022	Disease R-HSA-1643685	3.10E-43	200/1736
GO_Biological_Process_2021	Transmembrane receptor protein tyrosine kinase signaling	4.38E-43	95/404
KEGG_2021_Human	Phospholipase D signaling pathway	1.76E-42	59/148

Appendix 12  

**Table 18:** OCD tissue specificity: strongest tissue-gene associations. OCD: Obsessive-compulsive disorder.

Trait	Gene	Tissue	Z
OCD	ANK3	Brain frontal cortex BA9	4.0377
OCD	ANK3	Brain nucleus accumbens, basal ganglia	4.0377
OCD	ANK3	Brain caudate, basal ganglia	4.0377
OCD	ANK3	Brain anterior cingulate cortex BA24	4.0377
OCD	ANK3	Brain hippocampus	4.0377
OCD	GLS2	Brain nucleus accumbens, basal ganglia	-3.4822
OCD	GLS2	Brain caudate, basal ganglia	-3.4822
OCD	GLS2	Brain frontal cortex BA9	-3.4822
OCD	GLS2	Brain amygdala	-3.4822
OCD	GLS2	Brain hippocampus	-3.4822
OCD	ACO2	Brain caudate, basal ganglia	3.2862
OCD	HTR6	Brain hippocampus	2.6718
OCD	HTR6	Brain caudate, basal ganglia	2.6718
OCD	HTR6	Brain anterior cingulate cortex BA24	2.6718
OCD	HTR6	Brain frontal cortex BA9	2.6718
OCD	MAPK3	Brain frontal cortex BA9	2.6641
OCD	MAPK3	Brain amygdala	2.6641
OCD	MAPK3	Brain hippocampus	2.6641
OCD	MAPK3	Brain nucleus accumbens, basal ganglia	2.6641
OCD	DRD2	Brain nucleus accumbens, basal ganglia	2.5838
OCD	MAPK1	Brain caudate, basal ganglia	2.5373
OCD	MAPK1	Brain nucleus accumbens, basal ganglia	2.5373
OCD	ACSL6	Brain nucleus accumbens, basal ganglia	-2.3738
OCD	ACSL6	Brain anterior cingulate cortex BA24	-2.3738
OCD	ACO2	Brain nucleus accumbens, basal ganglia	2.3261

Appendix 13  

**Table 19:** Hub-gene tissue profile in OCD. OCD: Obsessive-compulsive disorder.

Gene	Peak Z	Peak tissue	Number of tissues
GABRA6	0.279	Brain caudate, basal ganglia	4
RPTOR	-0.342	Brain amygdala	4
MAPK3	2.664	Brain amygdala	6
GABRB2	0.25	Brain amygdala	3
GNB3	-1.925	Brain nucleus accumbens, basal ganglia	6
DRD2	2.584	Brain nucleus accumbens, basal ganglia	6
GSK3B	-1.465	Brain anterior cingulate cortex BA24	4
ANK3	4.038	Brain anterior cingulate cortex BA24	6
CYP2D6	-1.775	Brain hippocampus	4
CHRM5	1.143	Brain amygdala	1
HTR6	2.672	Brain anterior cingulate cortex BA24	4
CACNA1B	-0.572	Brain caudate, basal ganglia	1
MAPK1	2.537	Brain caudate, basal ganglia	3
SF3B1	0.488	Brain anterior cingulate cortex BA24	3
FYN	1.189	Brain anterior cingulate cortex BA24	5
LIMK1	0.388	Brain frontal cortex BA9	1
CTNNB1	-1.35	Brain hippocampus	1
GLS2	-3.482	Brain amygdala	6

Appendix 14  

**Table 20:** Prioritized genes by maximum absolute Z in OCD follow-up. OCD: Obsessive-compulsive disorder.

Gene	Maximum absolute Z	Z in OCD	Number of traits
ANK3	9.1227	9.1227	1
SNF8	8.6781	8.6781	1
GLS2	7.3297	-7.3297	1
CHRNB1	7.0753	-7.0753	1
LAMTOR3	6.8945	6.8945	1
VPS45	6.8845	-6.8845	1
LYNX1	6.8004	-6.8004	1
WASHC3	6.649	-6.649	1
PPP1CB	6.1154	6.1154	1
MAPK3	6.0639	6.0639	1
MAP1LC3A	5.96	5.96	1
PPP2CB	5.9375	-5.9375	1
C3	5.7397	-5.7397	1
VPS4A	5.7365	5.7365	1
VPS28	5.6555	-5.6555	1
MAP2K1	5.6464	-5.6464	1
GORASP2	5.5065	5.5065	1
PFN1	5.4595	-5.4595	1
BAD	5.4422	5.4422	1
ACO2	5.359	5.359	1
HTR6	5.3435	5.3435	1
ATG2A	5.293	5.293	1
METTL21A	5.2441	-5.2441	1
ATG10	5.2363	-5.2363	1
TAC3	5.2146	-5.2146	1

Appendix 15  

**Table 21:** DGIdb drug-ranking output: top drugs by number of leading-edge targets. Note. The DGIdb table is an annotation screen only and should not be interpreted as evidence of therapeutic suitability.

Drug	Number of targets	Mean score	Representative target genes
Sorafenib	38	0.042	ABCB1; ATXN2; BRAF; CBL; CSF1R; CTNNB1; DRD2; EGFR; EPHA2; FLT3
PF-562271	35	0.026	ACSL1; ACSL3; CAMKK2; DAPK3; EPHA2; FLT3; FLT4; FYN; GRK5; GSK3B
Cenisertib	31	0.013	AKT1; CSF1R; DAPK3; DRD2; EGFR; ERBB4; FLT3; FLT4; FYN; INSR
NVP-TAE684	29	0.015	CSF1R; DAPK3; EGFR; ERBB4; FLT3; FLT4; FYN; GRK5; INSR; IRAK4
Ilorasertib	28	0.012	CSF1R; DRD2; EGFR; EPHA2; ERBB4; FLT3; FLT4; FYN; INSR; IRAK4
RG-1530	27	0.014	CSF1R; DRD2; EGFR; EPHA2; ERBB4; FLT3; FLT4; FYN; GRK5; GSK3B
Dasatinib anhydrous	26	0.05	ABCB1; ABL2; BRAF; CBL; CRKL; CSF1R; EPHA2; ERBB4; FYN; GMNN
Dovitinib	26	0.024	CAMKK2; CFLAR; CSF1R; DRD2; FGFR4; FLT3; FLT4; FYN; INSR; LIMK1
Clozapine	25	0.047	A1BG; A2M; ABCF1; ALDH1A1; ALDH2; AMD1; C3; CBX1; CCKBR; CHRM1
Doxorubicin hydrochloride	25	0.025	ABCB1; APEX1; ATXN2; BAZ2B; BRAF; CASP3; CPT1A; CYP2D6; EHMT2; FLT3
CYC-116	25	0.017	AKT1; CSF1R; CYP2D6; DAPK3; EGFR; EPHA2; FLT3; FYN; GSK3B; INSR
Haloperidol decanoate	24	0.146	A1BG; ALDH1A1; ALDH2; AMD1; BAAT; COMT; CYP2D6; DRD2; DTNBP1
Olanzapine	23	0.055	A1BG; A2M; ADRA2B; AMD1; CCKBR; CHRM1; CHRM3; CHRM5; DRD2; DRD5
Cisplatin	23	0.029	ADH1C; ALDH1A1; APEX1; CASP3; CBX1; CGA; DDIT3; EGFR; EHMT2; FAS
Tamatinib	23	0.025	DAPK3; EGFR; EPHA2; ERBB4; FLT3; FYN; INSR; IRAK4; MAP3K10
SP-600125	23	0.015	ALDH1A1; CSF1R; CYP2D6; DAPK3; ERBB4; FLT3; FLT4; GSK3B; INSR
Linifanib	22	0.037	CSF1R; DRD2; EPHA2; FLT3; FLT4; INSR; IRAK4; MAP2K2; MAP3K20
Tozasertib	22	0.022	ABL2; EGFR; EPHA2; FLT3; FLT4; FYN; GMNN; INSR; IRAK4; LIMK1
Aspirin	20	0.145	ACE; AGAP3; AGT; CPT1B; CPT2; CYP2D6; DDIT3; F2R; FAS; ME3
Celecoxib	20	0.069	BRAF; CACNA1A; CACNA1B; CACNA1D; CACNA1E; CACNA1I; CACNA2D1
Cyclophosphamide anhydrous	19	0.057	ACTB; ADH1C; CBX1; CPT1A; CTNNB1; CXCR2; CYP2D6; EHMT2; FAS
Methotrexate	19	0.04	ADA; APEX1; CLTC; CPT1A; CYP2D6; EHMT2; FGFR4; FLT3; FOLR1; GSK3B
Doxepin hydrochloride	19	0.04	ABCB1; AKT1; ALDH1A1; AMD1; CES1; CHRM1; CHRM3; CHRM5; COMT
Gefitinib	19	0.033	ABCB1; BRAF; CRKL; CYP2D6; CYP3A5; EGFR; ERBB3; ERBB4; FLT4
PD-0166285	19	0.024	CSF1R; EGFR; FLT3; FYN; MAP2K1; MAP2K2; MAP4K4; MAPK14; MAPK8

Appendix 16  

**Table 22:** Overlap with selected known psychotropic or bipolar disorder-related drugs.

Drug	Leading-edge targets
Carbamazepine	CYP2D6; CYP3A5; HLA-A; HLA-B; HSPA1L; TNF
Lamotrigine	CYP2D6; HLA-C
Lithium	ABCB1; DRD2; FAS; FKBP5; GNRH1; HTR1B; MAPK14; NTRK2; NTS; RABEP1; TPH1
Olanzapine	A1BG; A2M; ADRA2B; AMD1; CCKBR; CHRM1; CHRM3; CHRM5; DRD2; DRD5; EIF2AK4; GNB3; HRH1; HTR1B; HTR1D; HTR1E; HTR3A; HTR6; IL1A; PPARG; PRKAA2; RABEP1; SLC1A1

Appendix 17  

**Table 23:** Signed-ranking re-analysis in OCD. OCD: Obsessive-compulsive disorder; NMN: Nicotinamide mononucleotide; KEGG: Kyoto Encyclopedia of Genes and Genomes; AntiDep_Genes: Antidepressant-related gene comparator; NES: Normalized enrichment score; dNES: Difference in normalized enrichment score; TCA: Tricarboxylic acid; PPAR: Peroxisome proliferator-activated receptor; MAPKMitogen-activated protein kinase.

Trait	NMN pathway set	Comparator	NES1	p1	NES2	p2	dNES	Verdict
OCD	KEGG_AUTOPHAGY_ANIMAL	AntiDep_Genes	1.0085	0.234883	-1.0273	0.173913	2.0358	not better
OCD	KEGG_ENDOCYTOSIS	AntiDep_Genes	0.9155	0.370815	-1.0273	0.173913	1.9428	not better
OCD	KEGG_RAS_SIGNALING_PATHWAY	AntiDep_Genes	0.9086	0.366817	-1.0273	0.173913	1.9359	not better
OCD	KEGG_FATTY_ACID_DEGRADATION	AntiDep_Genes	0.8923	0.323338	-1.0273	0.173913	1.9196	not better
OCD	KEGG_CITRATE_CYCLE_TCA_CYCLE	AntiDep_Genes	0.8675	0.337831	-1.0273	0.173913	1.8948	not better
OCD	KEGG_SYNAPTIC_VESICLE_CYCLE	AntiDep_Genes	0.8225	0.423288	-1.0273	0.173913	1.8498	not better
OCD	KEGG_NEUROACTIVE_LIGAND_RECEPTOR_INTERACTION	AntiDep_Genes	0.7933	0.514243	-1.0273	0.173913	1.8206	not better
OCD	KEGG_PPAR_SIGNALING_PATHWAY	AntiDep_Genes	-0.6415	0.465767	-1.0273	0.173913	0.3858	not better
OCD	KEGG_MAPK_SIGNALING_PATHWAY	AntiDep_Genes	-0.989	0.212894	-1.0273	0.173913	0.0383	not better
OCD	KEGG_REGULATION_OF_ACTIN_CYTOSKELETON	AntiDep_Genes	-1.0398	0.170415	-1.0273	0.173913	-0.0125	not better
